# Integrated Analysis of the Cecal Microbiome and Plasma Metabolomics to Explore NaoMaiTong and Its Potential Role in Changing the Intestinal Flora and Their Metabolites in Ischemic Stroke

**DOI:** 10.3389/fphar.2021.773722

**Published:** 2022-01-20

**Authors:** Huiting Lin, Shaoru Chen, Lin Shen, Tao Hu, Jiale Cai, Sikai Zhan, Jiayin Liang, Mingmin Huang, Minghua Xian, Shumei Wang

**Affiliations:** ^1^ School of Traditional Chinese Medicine, Guangdong Pharmaceutical University, Guangzhou, China; ^2^ Key Laboratory of Digital Quality Evaluation of Chinese Materia Medica of State Administration of TCM, School of Traditional Chinese Medicine, Guangdong Pharmaceutical University, Guangzhou, China; ^3^ Engineering and Technology Research Center for Chinese Materia Medica Quality of the Universities of Guangdong Province, Guangdong Pharmaceutical University, Guangzhou, China

**Keywords:** ischemic stroke, intestinal microbiota, metabolomics, microbial metabolites, pseudo-germ-free

## Abstract

Ischemic stroke (IS), as a leading cause of disability worldwide, affects intestinal bacterial communities and their metabolites, while recent discoveries have highlighted the importance of the intestinal microflora in the development of IS. Systematic investigations of complex intestinal bacterial communities and their metabolites during ischemic brain injury contribute to elucidate the promising therapeutic targets for IS. However, the associations between intestinal microbiota and related circulating metabolic processes in IS remained unclear. Hence, to identify the changed microflora and their metabolites in IS of NaoMaiTong (NMT), an effective clinical medication, we established the middle cerebral artery occlusion/reperfusion (MCAO/R) model using conventionalized and pseudo-germ-free (PGF) rats. Subsequently, we systematically screen the microflora and related metabolites changing in IS *via* an integrated approach of cecal 16S rRNA sequencing combined with plasma metabolomics. We found that NMT relied on intestinal flora to improve stroke outcome in conventionalized rats while the protection of NMT was reduced in PGF rats. Total 35 differential bacterial genera and 26 differential microbial metabolites were regulated by NMT. Furthermore, L-asparagine and indoleacetaldehyde were significantly negatively correlated with *Lachnospiraceae_UCG.001* and significantly positively correlated with *Lachnoclostridium*. Indoleacetaldehyde also presented a negative correlation with *Lactobacillus* and *Bifidobacterium*. 2-Hydroxybutyric acid was strongly negatively correlated with *Ruminococcus, Lachnospiraceae_UCG.001* and *Lachnospiraceae_UCG.006.* Creatinine was strongly negatively correlated with *Akkermansia*. In summary, the research provided insights into the intricate interaction between intestinal microbiota and metabolism of NMT in IS. We identified above differential bacteria and differential endogenous metabolites which could be as prebiotic and probiotic substances that can influence prognosis in stroke and have potential to be used as novel therapeutic targets or exogenous drug supplements.

## Introduction

Ischemic stroke (IS), a 80–85% proportion of all types of stroke, is characterized by loss of neurological function and long-term disability (Musuka et al., 2015). The current clinical treatments were limited by narrow therapeutic time windows and high costs (2005; Jauch et al., 2013). Therefore, it is urgent to develop new drugs for the treatments of IS. However, due to the complicated and unclear pathological mechanism of IS, it restricts the development of new drugs for the treatment of IS. A number of observations suggested that the gut flora conducts a life-long, bi-directional, and symbiotic relationship with the central nervous system via direct and indirect approaches, referred to as the brain-gut microbiota axis (O'Hara and Shanahan, 2006; Rhee et al., 2009). According to analysis the influence of gut microbiota on brain, the ecosystem had been recognized as an effective tool for improving the development and prognosis of IS (Benakis et al., 2016; Benakis et al., 2020). Accumulating clinical evidence suggested that IS patients suffer a variety of intestinal tract complications with dysbacteriosis which may exacerbate neuroinflammation and hinder recovery from stroke (Coggrave et al., 2014; Cheng et al., 2020). Meanwhile, the reestablishment of intestinal flora feeds back to relieve neuroinflammation and promote stroke prognosis through the communication pathways such as the immune system (Benakis et al., 2016; Sadler et al., 2020), endocranium (Houlden et al., 2016), nervous system (Kurita et al., 2020; Xu et al., 2020), and humoral routes (Benakis et al., 2016; Singh et al., 2016; Kurita et al., 2020; Lee et al., 2020). However, the complexity of the intestinal microbiota introduces a particular challenge to identify the optimal treatment for ischemic stroke. Current researches focused on changes in the community and function of the intestinal microbiome in stroke, while they lacked systemic physiological investigation of the relationship and changes mechanisms of the intestinal flora and intestinal metabolites after IS. Therefore, exploration of the role of gut bacteria changing in IS was particularly vital for the identification of novel treatments.

Additionally, stroke causes the change of a broad spectrum of metabolic products (Vojinovic et al., 2020). During the last 30 years, multiple reliable and sensitive microbial biomarkers specific to stroke have been identified using metabolomics. Such biomarkers fall into three primary pathophysiologic categories: those that were excitotoxic or neurotoxic (Schousboe, 2003; Min et al., 2015), those that cause oxidative stress (Kossi and Zakhary, 2000; Aad et al., 2010; Jung et al., 2011), or inflammatory factors (Wang et al., 2005; Min et al., 2015). Nevertheless, it is currently unclear how metabolic processes of gut microflora influence the development of stroke and there is a lack of a clear description of the metabolic processes in the gut microbiome or their mechanisms (Sidorov et al., 2019). Intricate interactions between microbiota and their metabolic products are central to the development and progression of stroke. Therefore, establishing differentiated intestinal flora and metabolites will contribute to the characterization of biochemical processes in ischemic brain tissue, which in turn help identify a safe and convenient therapy such as the combination of probiotics and beneficial metabolites.

NaoMaiTong (NMT), an effective treatment used clinically in IS for many years (Li et al., 2019; Zhang W. et al., 2020), is a multi-botanical drug preparation consisting of emodin, aloe-emodin, 3′-Methyoxy puerarin, Ligustilide, Senkyunolide, Ginsenoside Rg1 etc. (Fan et al., 2017). Although NMT had been shown to be effective in protecting against ischemic stroke, its effect was contradictory with low intracerebral exposure of its active ingredients. We conjectured that the mechanism of NMT for stroke may be related to the regulation of the intestinal microbiota and its metabolic processes. In the present study, we first undertook a systematic investigation of the relationship and mechanisms between the gut microbiome and their metabolic processes in ischemic stroke by 16S rRNA gene sequencing and plasma metabolomics.

## Materials and Methods

### Animals

Male Sprague-Dawley rats (250 ± 10 g) were purchased from the Guangdong Medical Laboratory Animal Center (Guangzhou, China) and were housed in autoclaved, ventilated cages in a controlled environment (temperature 25°C, humidity 50 ± 5%, 12 h daylight cycle) with autoclaved bedding and feed. The feed and water were provided sufficiently. All animal procedures were approved by the Animal Ethics Committee of Guangdong Pharmaceutical University.

### Establishment of the Rat Model of Cerebral Ischemia

Using Longa’s middle cerebral artery occlusion/reperfusion (MCAO/R) method with modifications (Longa et al., 1989), a rat model of ischemia/reperfusion was established under aseptic conditions, as far as possible (autoclaved suits and surgical instruments, clinical gloves, surgical mask). Each rat was anesthetized and immobilized on a surgical board prior to creating a longitudinal incision in the neck. The inner and outer parts of the sternocleidomastoid muscle were separated, while the left common carotid and external carotid arteries were isolated and ligated. The internal carotid artery was clamped with a micro-arterial clip. Without disturbing the pterygopalatine section of the maxillary artery, a monofilament was inserted into the internal carotid artery along the common carotid artery to prevent blood flow, which was then fixed in place using a suture, after which the incision was quickly closed. The monofilament was removed after 2 h of ischemia to induce reperfusion injury. The changes of cerebral blood flow (CBF) during the process of surgery were tested during surgery by Laser Speckle Flowgraphy system (Barzegar et al., 2021). All aspects of surgery were performed on the sham group, except for insertion of the monofilament. The rats were maintained at a suitable temperature until they were conscious.

### Establishment of Pseudo-Germ-Free (PGF) Model

Rats were randomly allocated to either an antibiotic (Abx) or vehicle (sterile water) treatment group 3 days prior to surgery (Wang et al., 2021). Rats in the Abx group were administrated four antibiotics in sterile water containing metronidazole (200 mg/kg, Aladdin), ampicillin (200 mg/kg, Aladdin), vancomycin (100 mg/kg, Aladdin), and neomycin (200 mg/kg, Aladdin) daily for 3 days to ensure that the gut microbiome was eliminated (Yang et al., 2020). The antibiotic mixture was mixed thoroughly prior to gavage. Fecal samples were collected daily during administration and the intestinal microbiome was monitored in real-time to ensure that it had been eliminated (Vijay-Kumar et al., 2010; Yan et al., 2020). A low level of fecal bacteria was an indication that the PGF model had been established completely after 3 days.

### Experimental Design

The procedure for creating the NaoMaiTong (NMT) extract had been described previously (Fan et al., 2017). Briefly, *Panax ginseng* C.A.Mey., (Araliaceae, *Ginseng Radix et Rhizome*) (126 g), *Rheum palmatum* L. (Polygonaceae, *Rheum palmatum L. Radix et Rhizome*) (126 g), *Pueraria montana* var*. lobata* (Willd.) Maesen and S.M.Almeida ex Sanjappa and Predeep, (Leguminosae, *Puerariae Lobatae Radix*) (84 g) and *Conioselinum anthriscoides “Chuanxiong”*, (Apiaceae, *Chuanxiong Rhizoma*) (84 g) were added into 1.8 L 60% ethanol to extract twice (1 h/time) at 90°C, then, the filtrates were merged and concentrated to ethanol-free. Finally, the concentrated solutions were freeze-dried (FD-1A-50 Vacuum Freeze Dryer, Biocool, Beijing, China). Total 130.07 g lyophilized powders were obtained and the extractum obtained rate was 30.97%. To identify the components of NMT, water dissolved sample of NMT and multi-standards mixed samples were detected by UPLC-Q-Exactive-Orbitrap MS (Thermo Fisher Scientific, Bremen, Germany) (Supplementary Figure S1). Rats were allocated into four groups, namely the sham group, middle cerebral artery occlusion (model) group, positive medicine (Nimodipine) group, and NMT decoction (NMT) group. The sham, nimodipine, and NMT groups were treated intragastrically with either autoclaved water, with nimodipine (12.34mg/1 Kg dissolved in water), or NMT extractum (0.96 g/Kg, approximately equal to 3.085 g/Kg botanical drugs) daily for 7 days following surgery, respectively. According to the clinical dose, the dose of NMT was determined by the previous dose-effect relationship study (Chen et al., 2012; Yang et al., 2017; Li et al., 2019). The weight of each rat was recorded daily.

Adopting the above PGF rats to construct MCAO/R model, rats were allocated into six experimental groups: sham, sham plus Abx, MCAO/R model, MCAO/R model plus Abx, NMT, or NMT plus Abx. The rats were studied to ascertain the pharmacodynamic effect of NMT on the PGF rats with ischemic stroke.

### Behavioral Score and Infarct Area

Neurological function and balance capacity were assessed using a modification of the Longa mothed (Longa et al., 1989) and the balance beam test (Vanti et al., 2021), respectively. The standards of neurological function score and beam balance test are listed in Supplementary Tables S2,S3. Furthermore, the brain was removed from the skull and quickly frozen following saline perfusion then sliced into 20 mm-thick coronal sections. The slices were stained with triphenyltetrazolium chloride (TTC) phosphate buffer for 15 min. The area of infarcted tissue was estimated using ImageJ software (ImageJ 1.4, NIH, United States ), then the integral calculated to estimate infarct volume.

### Histological Analysis

The rats were sacrificed at day 7 after surgery. The intestines and brain tissues were harvested, placed rapidly into 4% paraformaldehyde (PFA) for 24 h, mounted with gelatin then embedded in paraffin. The tissues were cut into 5 μm slices after which the sections were deparaffinized and stained with hematoxylin and eosin (Servicebio, G1003, Wuhan, China). Alcian blue (AB)/periodic acid-Schiff (PAS) staining (Servicebio, Wuhan, China) was used to assess goblet cells in the colon. The sections were dehydrated and covered with a coverslip after the addition of Depex mounting medium. Histological images were captured using a microscope (cellSens Standard).

### Western Blot Analysis

Colon tissues were lysed in RIPA lysis buffer with 1% phosphatase inhibitor cocktail and 1% protease inhibitor cocktail (MedChemExpress, New Jersey, United States), they were homogenized and centrifuged in 4°C. Protein concentrations were measured with a Pierce BCA protein assay kit (Thermo Fisher Scientific, Waltham, Massachusetts, United States). Electrophoresis of 40 μg protein was proceeded by SDS-polyacrylamide gel electrophoresis (SDS-PAGE) and transferred to a polyvinylidene difluoride (PVDF) membrane (Millipore, Bedford, MA, United States). The membranes were blocked with 5% non-fat milk and incubated with antibodies. The antibodies are as follow: ZO1 (21773-1-AP, 1:1000, PROTEINTECH GROUP), MMP9 (ab76003, 1:3000, Abcam, Cambridge, MA, United States), GAPDH (ab8245, 1:5000, Abcam, Cambridge, MA, United States), HRP-conjugated goat anti-rabbit IgG secondary antibodies (SA00001-2, 1:5000, PROTEINTECH GROUP). The membranes were visualized using an ECL Plus kit (Thermo Scientific, United States) and exposed to MiniChem610 (SAGECREATION, Beijing, China). Images were analyzed using ImageJ software (National Institutes of Health, Bethesda, MD, United States).

### 16S Ribosomal RNA Sequencing

Total bacterial DNA was isolated from the cecal microbiome of each rat sample aseptically to avoid contamination with exogenous microflora using a PowerSoil DNA isolation kit, in accordance with the manufacturer’s protocol. qPCR was performed using bacterial primer sets for the V3-V4 regions of the 16 S rDNA genes. After the final PCR samples were purified, they were standardized to equal DNA concentrations then sequenced using an Illumina HiSeq 2500 platform (Youssef et al., 2009; Caporaso et al., 2011).

### Sample Extraction and LC-MS Analysis for Metabolomics

A 100 μl aliquot of sample and 400 μl of extract solution (acetonitrile: methanol = 1: 1, containing isotopically-labeled internal standard) were mixed in an EP tube, then ultrasonicated, and incubated 1 h at −40°C temperature prior to centrifuged at 13,800 *g* for 15 min at 4°C. The supernatant was then retained and analyzed. Quality control (QC) samples were prepared by mixing equal aliquots of supernatants from all samples.

LC-MS/MS analyses were performed using an UHPLC system (Vanquish, Thermo Fisher Scientific) with a UPLC BEH Amide column (2.1 mm × 100 mm, 1.7 μm) coupled to Q Exactive HFX mass spectrometer (Orbitrap MS, Thermo). The mobile phase consisted of 25 mmol/L ammonium acetate and 25 ammonia hydroxides in water (pH = 9.75, A) and acetonitrile (B). The auto-sampler temperature was 4°C, and the injection volume was 2 μl.

The QE HFX mass spectrometer was used for its ability to acquire MS/MS spectra on information-dependent acquisition (IDA) mode in the control of the acquisition software (Xcalibur, Thermo). In this mode, the acquisition software continuously evaluates the full scan MS spectrum. The ESI source conditions were set as following: sheath gas flow rate as 30 Arb, Aux gas flow rate as 25 Arb, capillary temperature 350°C, full MS resolution as 60,000, MS/MS resolution as 7,500, collision energy as 10/30/60 in NCE mode, spray Voltage as 3.6 kV (positive) or −3.2 kV (negative), respectively.

### Statistical Analysis

Uparse software (Uparse v7.0.1001, http://www.drive5.com/uparse/) (Haas et al., 2011) was used to cluster all effective tags of the samples to allow a selection of representative sequences in operational taxonomic units (OTUs). The OTU sequences were annotated for species from which taxonomic information was obtained. The community composition of each sample was recorded at each classification level: kingdom, phylum, class, order, family, genus, and species. MUSCLE software (Version 3.8.31, http://www.drive5.com/muscle/) (Christian et al., 2013) was used to perform rapid multiple sequence alignment and obtain the phylogenetic relationship of all OTU representative sequences. Finally, data for each sample was normalized. Alpha and Beta diversity analysis was based on data following normalization.

Prioritization analysis was performed on the UPLC-MS data. Raw data were converted to mzXML format using ProteoWizard and processed using in-house software developed using the R programming environment based on XCMS peak detection, extraction, alignment, and integration (2006). An in-house MS2 database was then used for metabolite annotation.

The difference analysis between groups of Alpha diversity and Beta diversity will be conducted by parametric test and non-parametric test respectively by Tukey test and Agricolae package Wilcox test. The screening criteria for differential metabolites were: *p* value < 0.05 by T-test, and VIP >1; The VIP value is the variable projection importance of the first principal component of the OPLS-DA model. Correlation between the differential concentration of flora and metabolomics metabolite data was calculated using a Spearman algorithm. The correlation coefficient (Corr) and *p* value (*p* value) matrices were used for subsequent analysis and for plotting graphs.

All statistical analyses were performed using GraphPad Prism software (GraphPad, version 8.0). The comparison was analyzed by t-test, one-way ANOVA, or two-way ANOVA followed by Tukey’s post hoc test. A *p* value < 0.05 was regarded as statistically significant.

## Results

### Neurological Function and Infarct Area Are Improved by NMT in Post-stroke Restoration

**FIGURE 1:**
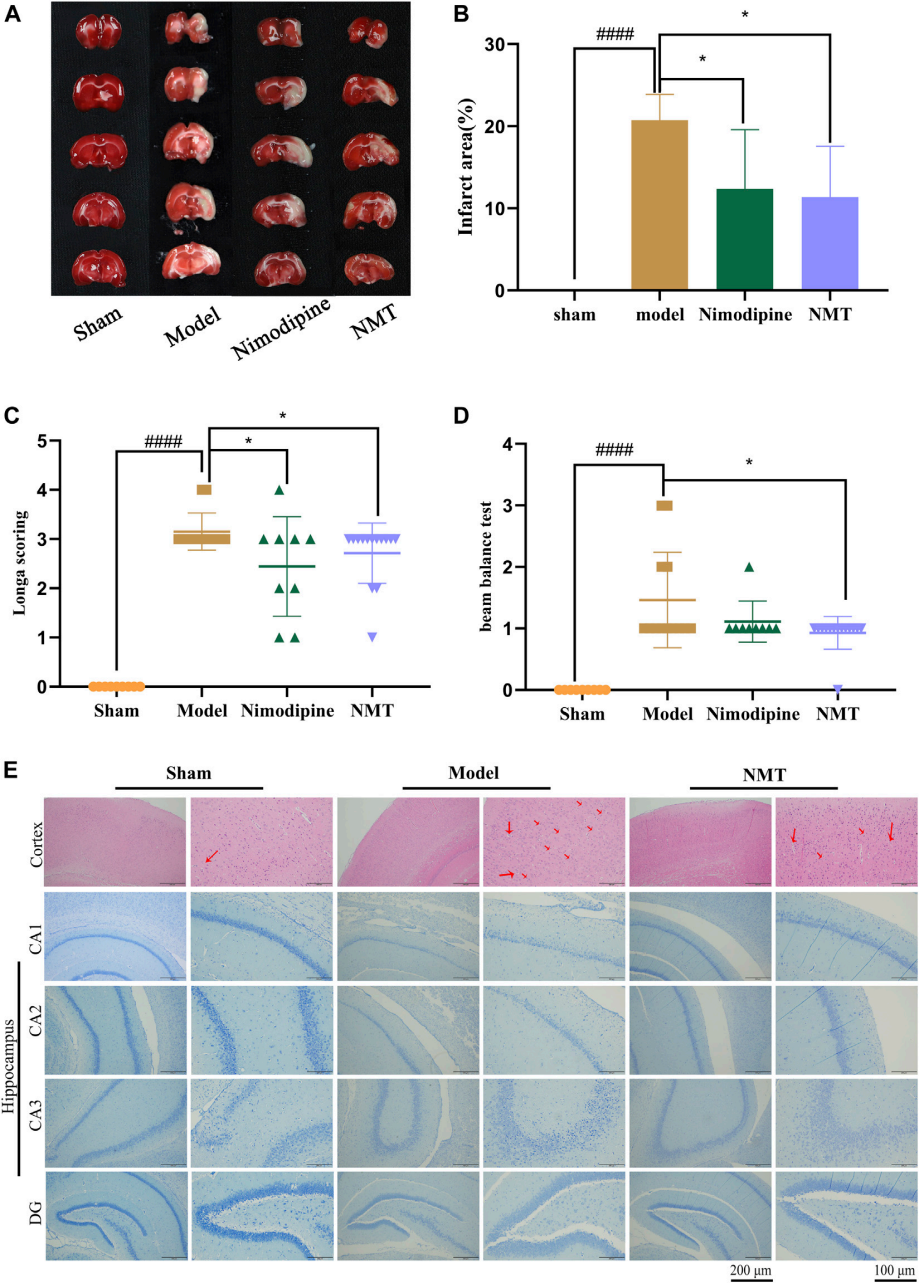
NMT improved neurological function and infarct area in post-stroke restoration. **(A)** TTC staining. **(B)** Cerebral infarct area percentage (*n* = 6). Compared with sham group, statistical significance was considered at ##*p* < 0.01, ###*p* < 0.001 for all the test; Compared with model group, statistical significance was considered at *p < 0.05, **p < 0.01 and ***p < 0.001 for all the tests. **(C)** Neurological score (*n* = 9). **(D)** Beam balance test (*n* = 9). Throughout, error bars represent mean ± SD. **(E)** H&E Staining of cerebral cortex and Nissl’s staining of cerebral hippocampus difference partition such as CA1, CA2, CA3, DG. The red arrows represent apoptotic neurons. (Scales bar = 200 and 100 μm *n* = 4).

The efficacy of NMT decoction in the therapy of cerebral ischemic stroke was assessed by neurological and behavioral function and cerebral infarction area. The results of TTC staining demonstrated that the brain hemispheres in sham group were symmetrical and stained red, while significant edema and white infarct area was observed on the infarcted side of the brains in the model group. Moreover, the size of cerebral infarction in NMT group was much smaller (Figure 1A). Quantification of TTC staining (Figure 1B) showed that the proportion of infarcted tissue was 28.74 ± 2.88% in the model rats, demonstrating that the model had been established successfully. The changes of cerebral blood flow (CBF) during the process of surgery were supplemented (Supplementary Figure S3). After administration of nimodipine or NMT, the infarction decreased to 12.36 ± 6.59% or 11.36 ± 5.67%, respectively. In addition, the ability of rats to balance and spontaneous movement was assessed using neurological scores and the balance beam test. The score of neurological function and beam balance test of the model rats were significantly lower than the sham rats, but significantly enhanced after NMT administration which indicated that NMT improved the spontaneous exercise and balance capability of model rats (Figures 1C,D).

Moreover, the number and morphology of neuronal cells in the cerebral cortex and hippocampus were assessed. Staining of the cerebral cortex demonstrated that nerve cells from the brains of model rats exhibit abnormal morphology with unclear nuclear boundaries with karyopyknosis and cell membranes. Using histopathology, fewer apoptotic neurons were observed in the NMT treatment group, which had more closely arranged and intact morphological characteristics than the model group. Histological analysis of the hippocampus emerged the same trend (Figure 1E) indicated that NMT protecting neurons and enhancing prognosis in stroke. These results indicate that NMT and nimodipine were efficacious in reducing cerebral infarction, while NMT also improved neurological function.

### Damage to the Intestinal Barrier Is Reversed by NMT

**FIGURE 2:**
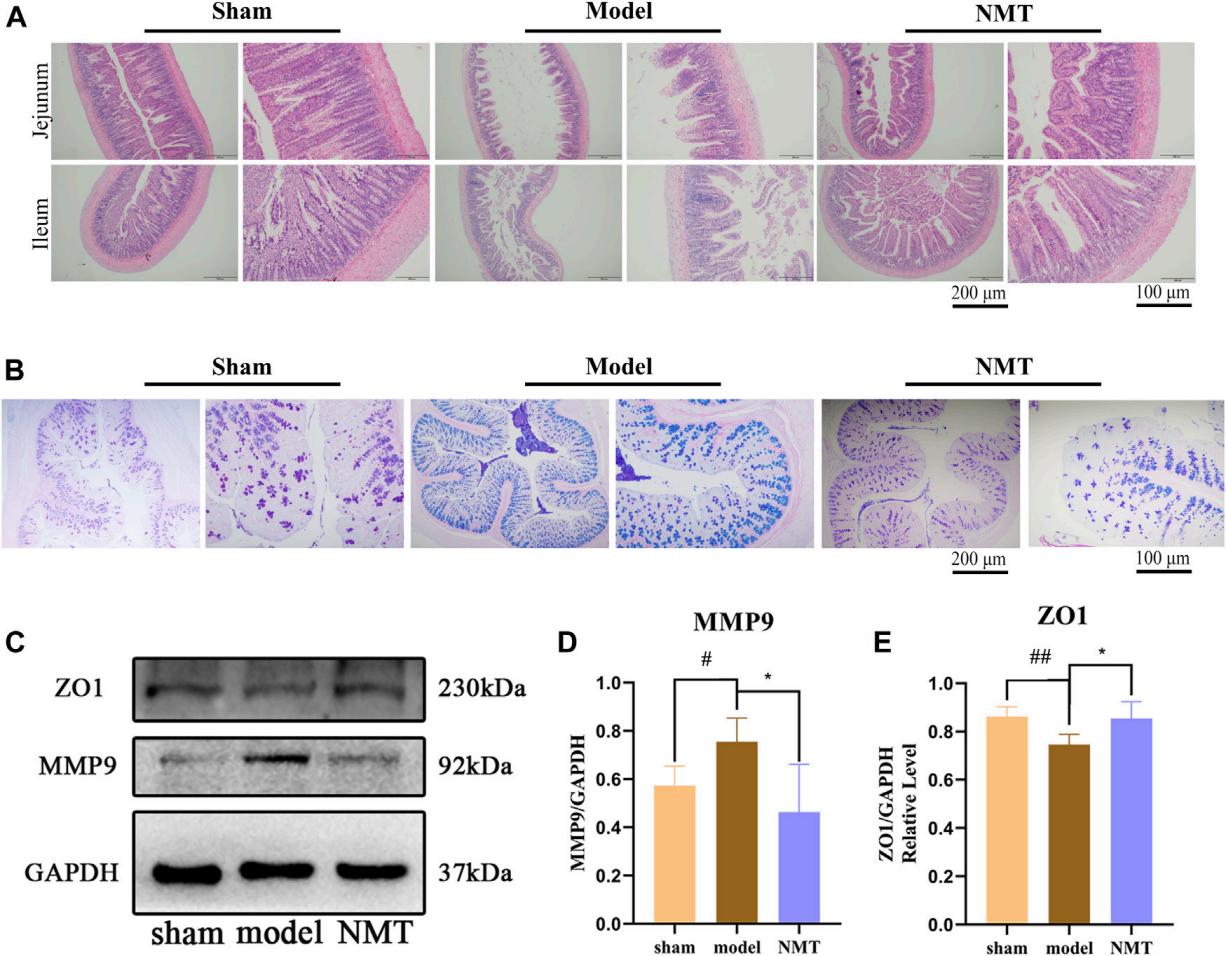
NMT reversed the Damage of the intestinal barrier. **(A)** H&E Staining of intestinal tissue (scale bar = 200 and 100 μm *n* = 4). **(B)** AB-APS Staining of intestinal tissue (scale bar = 200 and 100 μm *n* = 3). Goblet cells are purplish red. **(C)** Representative Western blot bands of different groups and **(D,E)** quantitative analyses of endogenous ZO1 and MMP9 in colon of different groups. (*n* ≥ 3). #*p* < 0.05, ##*p* < 0.01 compared with sham group; *p < 0.05, compared with the model group. Error bars represent mean ± SD.

Generally, the thin mucus layer and mucus layerlow expression of connexin were considered the symbol for the intestinal barrier injury. Changes in the intestinal barrier will affect the intestinal flora structure and its metabolites, such as leakage of endotoxins, migration and proliferation of pernicious bacteria (Lupp et al., 2007; Zhang P. et al., 2020). According to H&E staining, the intestinal epithelial cells of sham rats displayed complete villi structure and were tightly arranged. Unlike the typical morphology of the intestines in the sham rats, model rats displayed villi atrophy, crypt distortion, mucosal edema, and massive inflammatory cell infiltration. Particularly, compared with the model group, the extent of inflammation and damage in the NMT group was reduced, intestinal mucous layer was thickened, and the number of villi had recovered (Figure 2A). According to AB-APS staining, the number of goblet cells was significantly reduced in the model rats. Compared with the model rats, the number of goblet cells was increased in the administrated group signally (Figure 2B). These results were indicated that NMT had the protective effect on intestinal chemical barrier.

In comparison with the sham group, the expression of ZO-1, a tight junction (TJ) proteins and MMP9 (Zeng et al., 2021), a proteases responsible for the breakdown of intercellular connections, were observably changed in the model rats (*p* < 0.05 or *p* < 0.01). Compared with the model rats, the expressions of ZO-1 and MMP9 were observably recovered (*p* < 0.05), indicating that the intestinal barrier were destroyed suffered MCAO/R, while NMT had the protective effect on intestinal physical barrier (Figures 2C–E). Furtherly, the damage of intestinal barrier will destroy the homeostasis of intestinal flora to lead the proliferation and transfer of destructive bacteria, and the regulation of the intestinal flora will also promote the repair of intestinal barrier (Bolsega et al., 2019; Chen et al., 2021). Therefore, we speculated that homeostasis of intestinal flora was disequilibrate in the model rats and NMT recovered it by protected the intestinal microbial barrier.

### Post-stroke Restoration Is Related to the Reorganization of the Gut Microbiota

**FIGURE 3:**
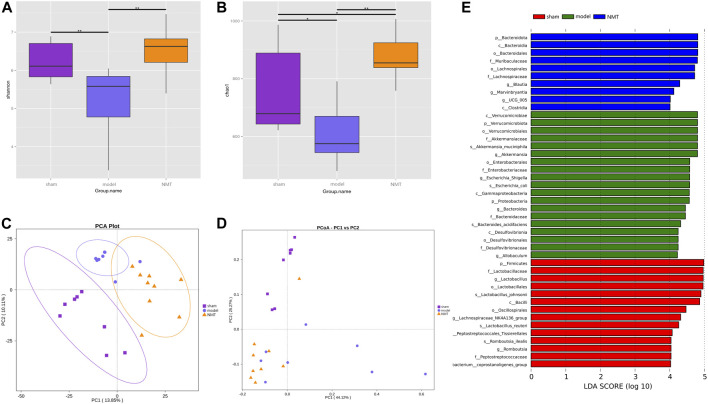
The adjustment of the structure of the intestinal flora was beneficial to the prognosis of stroke. **(A,B)** The Shannon index and chao1 index of α diversity. **(C,D)** Analysis of β diversity of intestinal flora in rats. PCA analysis and Weighted UniFrac PCoA analysis of gut microbiota based on the OTU data of sham, model, and NMT groups. Each point represents a sample. A clear separation is observed between the samples of sham (*n* = 8), model (*n* = 7) and NMT (*n* = 9) groups. **(E)** LEfSe analysis of different groups. Biomarkers at the genus level for each group screened based on LDA score. Different colors represent different groups. All group comparison in figures were analyzed with Kruskal–Wallis test.

To determine the influence of stroke on intestinal microflora, the cecal contents of intestinal microflora were sequenced in rats of different groups by 16S rRNA gene. The Shannon and Chao1 indices, indicators of bacterial diversity, richness and uniformity of intestinal microbial communities, indicated diverse outcomes in three groups which significantly decreased in the model group rats while consistently reversed in NMT group (Figures 3A,B). Furthermore, principal component analysis (PCA) and principal coordinate analysis (PCoA) of OTUs revealed a conspicuous separation of clusters of gut microbes of different groups of rats, indicating a reorganization of intestinal microbial communities after stroke and treatment (Figures 3C,D). The Anosim analysis between different groups were listed (Supplementary Figure S5).

Linear discriminant analysis (LDA) of effect size is an analytical tool for identifying and explaining high latitude data biomarkers, highlighting the statistically different biomarkers between different groups (Nicola Segata et al., 2011). Present study demonstrated that NMT remodeled the intestinal microbiota by enriching salutary endogenous bacteria. A greater number of pernicious bacteria and opportunistic pathogens, such as *Akkermansiaaceae, Enterobacteriaceae, and Desulfuribriaceae,* were enriched in the model group. Conversely, beneficial bacteria, such as *Bacteroidaceae, Muribaculaceae, Ruminococcaceae,* and *Lachnospiraceae,* were enriched following administration of NMT decoction (Figure 3E). The results revealed that pathogenic and opportunistic pathogenic microbes were enriched as the process of stroke developed, while the microbiota was remodeled *via* enrichment of the advantageous endogenous bacteria during the resolution of stroke symptoms.

**FIGURE 4:**
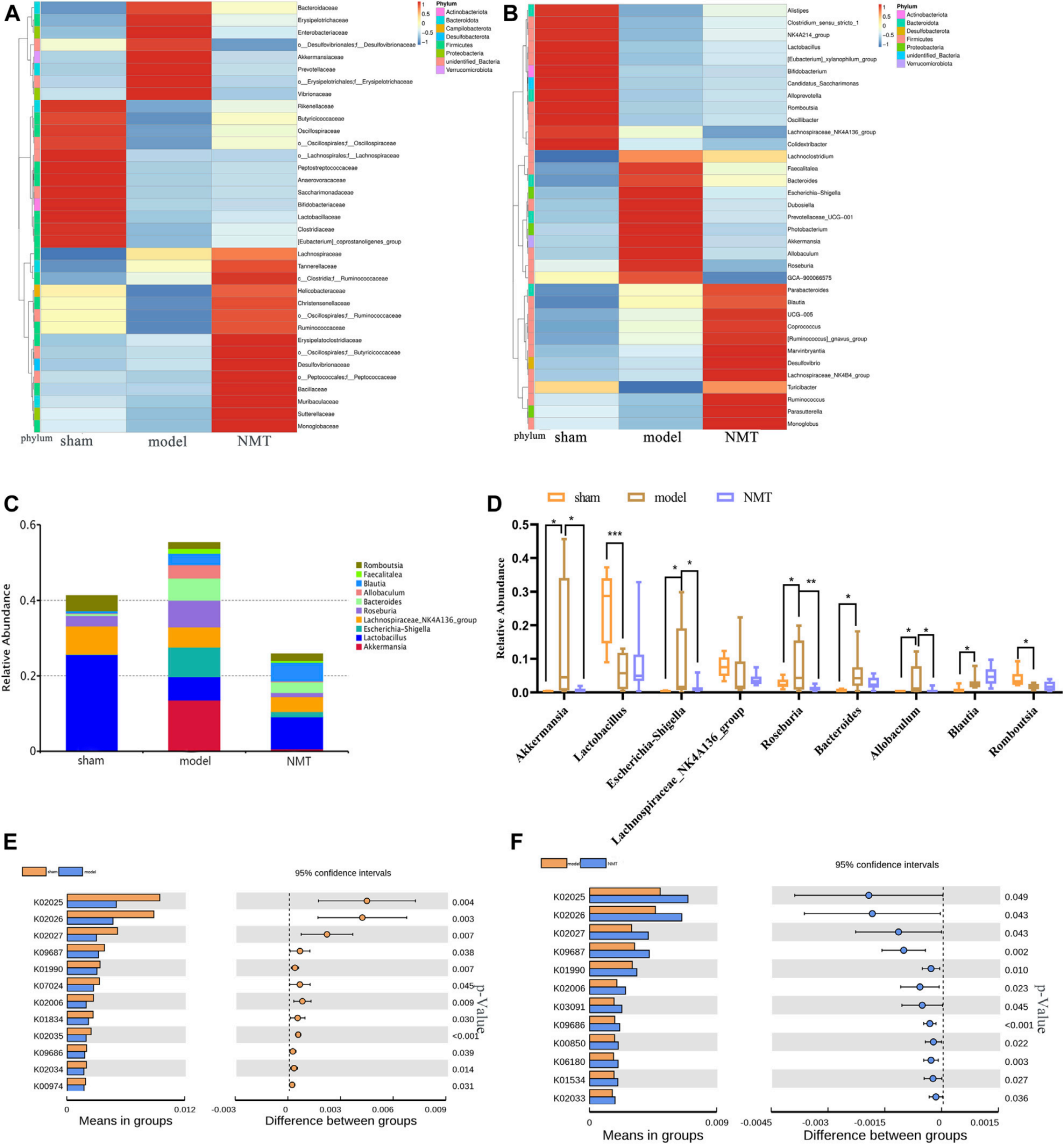
Stroke recovery was related to the increase of beneficial bacteria in the intestinal flora. **(A)** Heatmap of the relative abundances of the 35 most abundant intestinal bacteria which significantly changed in stroke (*n* = 7) compared with those in sham (*n* = 8) and NMT (*n* = 9) group rats at the level of the family (FDR-adjusted *p* < 0.05, FDR < 5%). The color bar indicates Z score that represents the relative abundance. Z score < 0 and >0 means the relative abundance is lower and higher than the mean. **(B)** Heatmap of the 35 most abundant bacterial genera, whose abundances changed significantly in different group rats. The color bar with numbers indicates the correlation coefficients. **(C)** The relative abundance of the gut bacterial phylum in each group **(D)** The relative abundances of the 9 most abundant bacterial genera that significantly correlated in different groups. Statistical significance was considered at *p < 0.05, **p < 0.01 and ***p < 0.001 for all the tests. **(E)** Predicted metabolic pathways using PICRUSt analysis with Student’s t test (*p* < 0.05).

The correlation between bacterial abundance and stroke prognosis was analyzed to reveal the pathological influence of the substantial modification of the gut microbiome induced by stroke. At a family level, there were 35 gut bacteria significantly changed in model rats compared with sham rats, of which 21 were substantially reduced (Figure 4A), while NMT increased the abundance of 26 bacteria and decreased the abundance of 9 bacteria compared with model rats. At a more detailed taxonomic level, 16 genera decreased in model rats, mainly including *Firmicutes*, *Bacteroidetes* and *Actinobacteria*, while 19 genera were more abundant, principally belonging to *Bacteroidetes, Desulfobacteria, and Proteobacteria* (Figure 4B). Thus, in rats experiencing a stroke, there were fewer potential pathobionts following gavage with NMT. Furthermore, three most abundant bacteria in the microbiome after induction of a stroke were *Akkermansia, Escherchia-Shigella, and Roseburia*, suggesting that they may play a critical role in stroke pathogenesis (Figures 4C,D). Additionally, the bioinformatics software package PICRUSt was used to determine the functional composition of the metagenome for different groups using taxonomic data. Analysis showed that numerous metabolic pathways and functions of the gut microbiome, including multiple sugar transport system permease protein, antibiotic transport system ATP-binding protein, and ABC-2 type transport system ATP-binding protein, were significantly enriched in the NMT group, indicating that favorable stroke prognosis was strongly associated with the system proteins above (Figures 4E,F). The pathways and their relative abundance of each group represented by KEGG Orthology (KO) were supplemented (Supplementary Table S5,S6).

### NMT Significantly Changed the Metabolic Profiles of Gut Microbiome in Rats After Stroke

**FIGURE 5:**
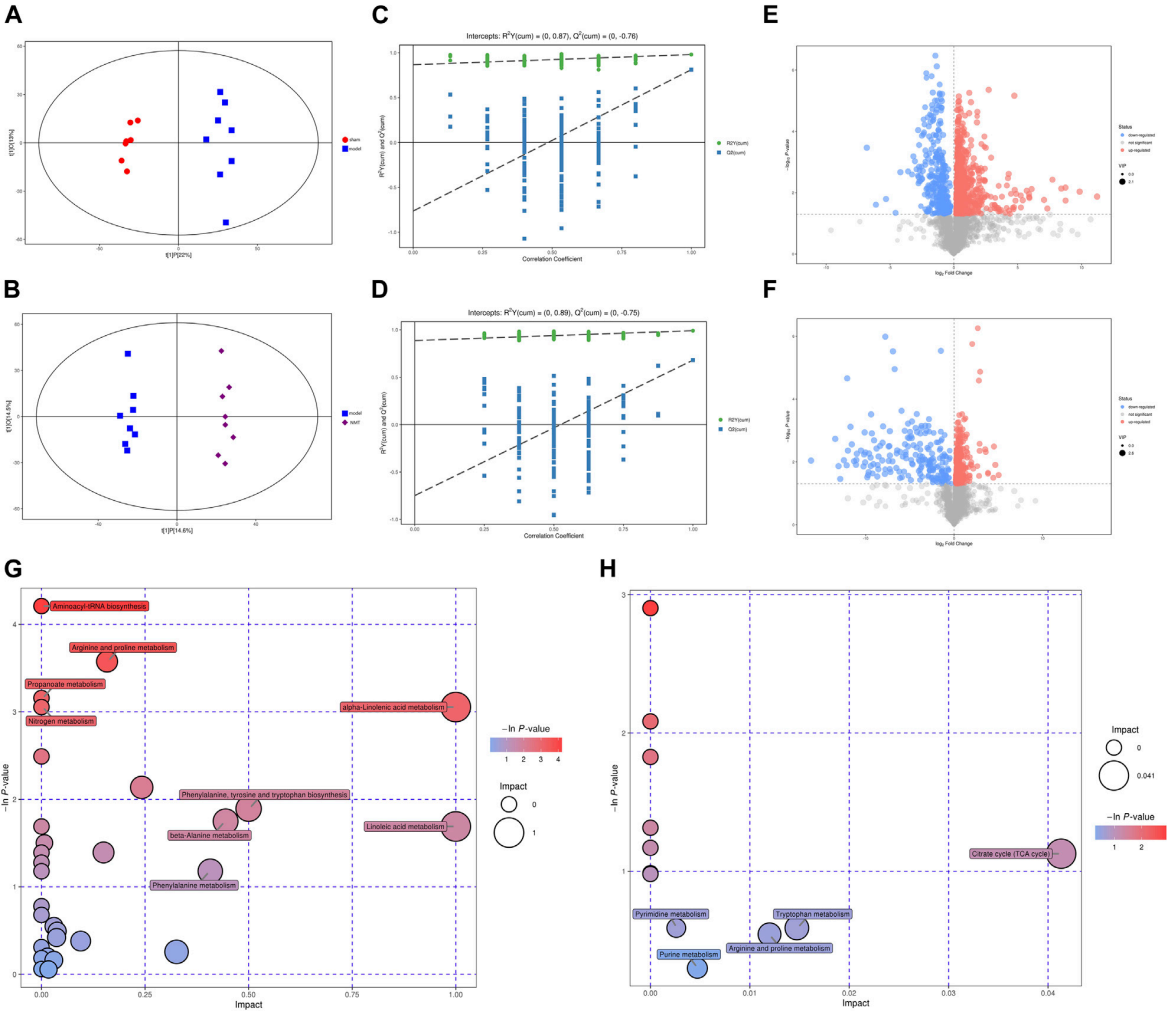
The significant changes in metabolic profiles of gut microbiota in stroke and recovered rats. **(A)** The plots of OPLS-DA scores of all peak features in negative (ES-) ion modes from the untargeted metabolomics analysis of stool samples of the rats in sham (*n* = 7) and model (*n* = 8) groups; **(B)** The plots of OPLS-DA scores of all peak features in negative (ES-) ion modes of the rats in model (*n* = 8) and NMT (*n* = 8) groups; **(C)** Negative scatter plots of the statistical validations obtained by 200X permutation tests in sham (*n* = 7) and model (*n* = 8) groups. R2 measures the goodness of fit and Q2 measures the predictive ability of the model. The criterion for model validity is that the regression line of the Q2-points (blue dotted line) intersects the vertical solid line (on the left) below zero; **(D)** Negative scatter plots of in model (*n* = 8) and NMT (*n* = 8) groups; **(E)** Negative volcano plot; volcano plots of the peak features of intestinal metabolites which significantly changed in three group in negative ion mode (ES-, upper panel). Red and blue circles indicate the significantly increased and decreased metabolites, respectively, (VIP >1, *p* < 0.05) in model VS sham group. The color tone indicates *p* value: a dark color indicates a small *p* value. The circle radius indicates the VIP value of corresponding peak features. **(F)** Negative volcano plot in model VS NMT group; **(G)** Bubble plot of important metabolic pathways for model VS sham group, as identified using KEGG pathway enrichment analysis; The size of the circle represents the impact factor obtained by topological analysis, the color tone indicates ln*p* value; **(H)** Bubble plot of important metabolic pathways for model VS NMT group.

As a bridge between microbiome and host, metabolites of the gut microbiota can impact host physiological status both within the gut and after entering the bloodstream. Thus, plasma samples from different groups were analyzed through metabolic profiling by HPLC-MS. A total of 4,296 and 4,382 peaks were recognized in negative and positive ion modes, respectively. The peaks were clustered by orthogonal partial least squares discriminant analysis (OPLS-DA) to obtain more reliable information about the data on differences between metabolite groups and correlation with the experimental groups, and further test the validity of the model. The results displayed metabolic data clusters of different groups separated from each other, indicating the presence of a number of different potential biomarkers (Figures 5A,B; Supplementary Figures S6A,B). Explanation and verification of the model were obtained after 200X permutation tests of OPLS-DA analysis, for which *R* ^2^ = 0.87, Q^2^ = −0.76; and *R* ^2^ = 0.89, Q^2^ = −0.75 for the model vs sham group and model vs NMT group in negative ion mode, respectively, and *R* ^2^ = 0.89, Q^2^ = −0.68; and *R* ^2^ = 0.82, Q^2^ = −0.38 for the model vs sham group and model vs NMT group in positive ion mode, respectively, indicating that the model adequately explained differences between the two groups of samples (Figures 5C,D; Supplementary Figures S6C,D).

Additionally, the differential metabolites were filtered and plotted using OPLS-DA. A total of 1,313 and 726 peaks with an effective change in peak intensity in ES- mode for the model vs sham group and model vs NMT group, respectively; a total of 936 and 202 peaks changed in ES + mode for model vs sham group and model vs NMT group, respectively (Figures 5E,F; Supplementary Figures S6E,F). The peaks above were analyzed by tandem mass spectrometry, from which multiple metabolites were identified by combining precise molecular weights and obtaining structural information from compound structure databases. We identified 88 host different metabolites in model rats which included 39 gut-derived metabolites and 46 host different metabolites in administrated rats which included 26 gut-derived metabolites. The different gut-derived metabolites were listed in Table 1, Table 2. These metabolites were produced or controlled directly by the gut microbiome, indicating that ischemic stroke significantly imbalanced gut microbiome metabolic profiles, while NMT restored them. Using comprehensive analyses (including enrichment and topological analysis) of pathways where differential metabolites were located, we can further screen the pathways and identify the critical pathways (Figures 5G,H). Furthermore, enrichment analysis of plasma metabolites indicated that there were important metabolic pathways including metabolism of linolenic acid, phenylalanine, tyrosine and tryptophan, beta-alanine, and nitrogen in model rats; citrate cycle and tryptophan metabolism in administrated rats.

**TABLE 1:** The 39 different gut-derived metabolites of MCAO/R male rats were obtained from the negative ion scan modes.

Metabolites	RT	Quant mass	VIP	*p*-value	Log_2_(FC)
Sphingosine 1-phosphate	310.29	378.24[M-H]-	1.12	3.77E-02	−0.57
Linoleic acid	63.61	279.23[M-H]-	1.14	4.81E-02	−0.69
N-Acetylaspartylglutamic acid	456.2	303.08[M-H]-	1.16	3.80E-02	1.14
Ascorbic acid	93.64	175.02[M-H]-	1.18	1.38E-02	0.91
N-Acetylornithine	377.57	173.09[M-H]-	1.21	7.68E-03	−0.7
1,3,5-Trihydroxybenzene	322.64	125.02[M-H]-	1.23	2.60E-02	−0.26
Terephthalic acid	395.93	165.02[M-H]-	1.24	2.46E-02	0.34
N-Formyl-L-aspartate	410.2	160.02[M-H]-	1.24	2.14E-02	0.31
Creatinine	182.8	112.05[M-H]-	1.29	1.30E-02	0.29
L-Glutamine	393.03	145.06[M-H]-	1.29	2.41E-02	0.19
L-Proline	330.94	114.06[M-H]-	1.3	3.48E-02	0.48
beta-Alanine	321.42	88.04[M-H]-	1.31	3.65E-02	0.66
Imidazoleacetic acid	290.95	125.03[M-H]-	1.32	2.08E-02	0.26
Dodecanoic acid	49.09	199.17[M-H]-	1.35	3.37E-02	−0.67
L-Phenylalanine	279.35	164.07[M-H]-	1.35	1.17E-02	0.63
1H-Indole-3-acetamide	48.56	173.07[M-H]-	1.35	3.16E-02	0.86
L-Methionine	304.47	148.04[M-H]-	1.36	1.42E-02	0.52
Cortisone	149.82	359.19[M-H]-	1.37	3.18E-02	−1.98
L-Asparagine	395.75	131.05[M-H]-	1.39	1.83E-02	0.5
Cytidine	260.52	242.08[M-H]-	1.46	1.67E-02	−0.75
5-Aminopentanoic acid	320.56	116.07[M-H]-	1.49	1.07E-02	0.7
Indoleacetaldehyde	62.75	158.06[M-H]-	1.5	7.13E-03	0.59
*m*-Coumaric acid	56.69	163.04[M-H]-	1.5	1.85E-02	0.95
D-Glucose	238.49	179.06[M-H]-	1.52	9.83E-03	0.41
2-Pyrocatechuic acid	25.4	153.02[M-H]-	1.52	1.83E-02	2.09
Creatine	34.32	130.07[M-H]-	1.52	3.48E-03	0.6
*p*-Hydroxyphenylacetic acid	49.57	151.04[M-H]-	1.54	4.23E-03	0.86
Phthalic acid	374.2	165.02[M-H]-	1.55	4.97E-03	0.22
2-Hydroxybutyric acid	205.48	103.04[M-H]-	1.56	3.40E-03	0.73
L-Arginine	534.51	173.1[M-H]-	1.56	6.66E-03	0.27
Hydroxypropionic acid	202.85	89.02[M-H]-	1.6	4.26E-03	0.34
Perillic acid	60.81	165.09[M-H]-	1.6	9.91E-04	−1.15
Alpha-Linolenic acid	42.06	277.22[M-H]-	1.61	9.78E-06	−1.3
L-Histidine	395.71	154.06[M-H]-	1.71	6.28E-04	0.51
Arachidonic acid	38.79	303.23[M-H]-	1.74	6.33E-06	−1.1
Taurocholic acid	219.93	514.29[M-H]-	1.75	3.46E-02	4.67
3-(3-Hydroxyphenyl)propanoic acid	171.63	165.05[M-H]-	1.87	8.10E-03	4.77
Mesaconic acid	208.35	129.02[M-H]-	1.88	4.18E-03	2.41
Gentisic acid	66.15	153.02[M-H]-	2.04	6.87E-06	4.73

**TABLE 2:** Effect of NaoMaiTong (NMT) on 26 gut-derived metabolites of MCAO/R male rats were obtained from the negative ion scan modes.

Metabolites	RT	Quant mass	VIP	*p*-value	Log_2_(FC)
Sinapyl alcohol	107.14	209.08[M-H]-	1.04	2.59E-02	−1.84
Indoleacetaldehyde	62.75	158.06[M-H]-	1.21	4.79E-02	0.45
L-Asparagine	395.75	131.05[M-H]-	1.23	4.74E-02	0.38
Creatine	34.32	130.07[M-H]-	1.36	6.00E-03	0.71
Pseudouridine	262.37	243.06[M-H]-	1.38	2.89E-02	0.3
Gluconolactone	63.67	177.04[M-H]-	1.41	3.63E-02	0.51
LysoPA(16:0/0:0)	220	409.24[M-H]-	1.44	3.33E-02	0.36
Hydroxypropionic acid	202.85	89.02[M-H]-	1.47	2.13E-02	0.3
N-Formyl-L-aspartate	410.2	160.02[M-H]-	1.51	1.71E-02	0.32
L-Threonine	371.05	118.05[M-H]-	1.51	1.31E-02	0.43
Adenine	318.95	134.04[M-H]-	1.54	2.10E-02	0.6
alpha-Ketoisovaleric acid	67.13	115.04[M-H]-	1.55	1.60E-02	0.26
Isocitric acid	97.66	191.02[M-H]-	1.55	3.39E-02	0.78
N-Acetylarylamine	210.03	134.06[M-H]-	1.56	3.28E-02	−0.95
Hippuric acid	210.22	178.05[M-H]-	1.57	2.69E-02	−0.99
Deoxycytidine	225.58	226.08[M-H]-	1.6	1.36E-02	0.41
Creatinine	182.8	112.05[M-H]-	1.68	6.39E-03	0.41
3-Hydroxybutyric acid	376.24	103.04[M-H]-	1.74	4.33E-03	0.54
Capric acid	40.58	171.14[M-H]-	1.76	3.77E-03	0.4
Oleic acid	348.84	281.25[M-H]-	1.77	6.78E-03	0.62
2-Hydroxybutyric acid	205.48	103.04[M-H]-	1.82	2.50E-03	0.76
trans-Cinnamic acid	121.1	147.04[M-H]-	1.82	4.17E-03	−1.63
Phenylglyoxylic acid	54.89	149.02[M-H]-	2.01	1.40E-03	−1.07
Deoxycholic acid	171.1	391.29[M-H]-	2.02	1.64E-02	2.17
Citraconic acid	120.01	129.02[M-H]-	2.1	2.58E-05	2.8
*Ortho*-Hydroxyphenylacetic acid	35.25	151.04[M-H]-	2.28	2.92E-06	−1.49

### Association Analysis of Microbial Diversity With Plasma Metabolites

**FIGURE 6:**
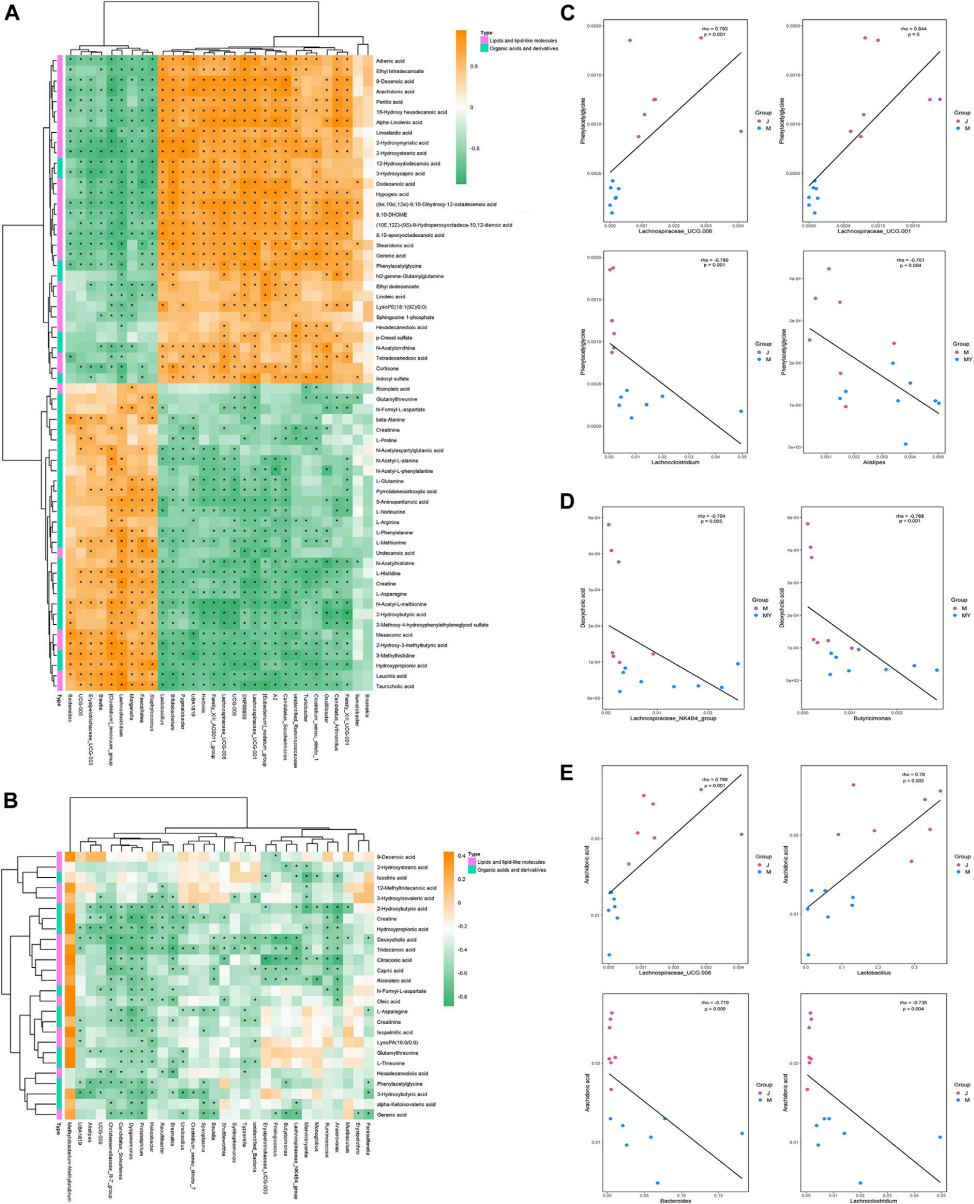
The correlation between gut microbiota and metabolites in development and outcome of stroke. **(A)** Heatmap of Spearman correlations between the bacteria whose abundances significantly changed in model (*n* = 8) VS sham (*n* = 7) group and the 2 type of metabolites (lipids and lipid-like molecules and organic acids and derivatives) with important functions and significant differences. The color bar with numbers indicates the correlation coefficients; **(B)** Heatmap of Spearman correlations between the discrepant bacteria in model (*n* = 8) VS NMT (*n* = 8) group and the 2 type of metabolites; **(C)** Relationship between intestinal L-asparagine level and *Lachnospiraceae_UCG.001* and *Lachnoclostridium* whose abundances significantly changed in model rats. rho: the spearman correlation coefficient; p: statistical significance. *p* < 0.05 and |rho| > 0.7; **(D)** Relationship between intestinal Indoleacetaldehyde level and the four most changed abundant bacterial genera; **(E)** Relationship between intestinal 2-Hydroxybutyric acid and Creatinine level and the most changed abundant bacterial genera.

The metabolites and associated enzymes which secreted and carried from the gut microbiome participating in biotransformations, have been investigated largely in relative isolation, but the overarching role of the gut microbe community in these biochemical reactions remains mostly uncharacterized (Wikoff et al., 2009). To identify the important metabolite processes in the development and prognosis of IS, the significantly different metabolites, lipids, and lipid-like molecules, and organic acids and derivatives were screened and visualized. Spearman correlation coefficients were computed between differential gut microbiome and distinct metabolites (Figures 6A,B). L-asparagine and indoleacetaldehyde were significantly negatively correlated with *Lachnospiraceae_UCG.001* and significantly positively correlated with *Lachnoclostridium*. Indoleacetaldehyde also presented the negative correlation with *Lactobacillus* and *Bifidobacterium*. 2-Hydroxybutyric acid was strongly negatively correlated with *Ruminococcus, Lachnospiraceae_UCG.001* and *Lachnospiraceae_UCG.006.* Creatinine was strongly negatively correlated with *Akkermansia* (Figures 6C–E). Considering the above relationship, it is reasonable to consider that the prognosis of stroke may be associated with changes in gut microbiome and their metabolites.

### Efficacy of NaoMaiTong was Reduced in Stroke Without the Gut Microflora

**FIGURE 7:**
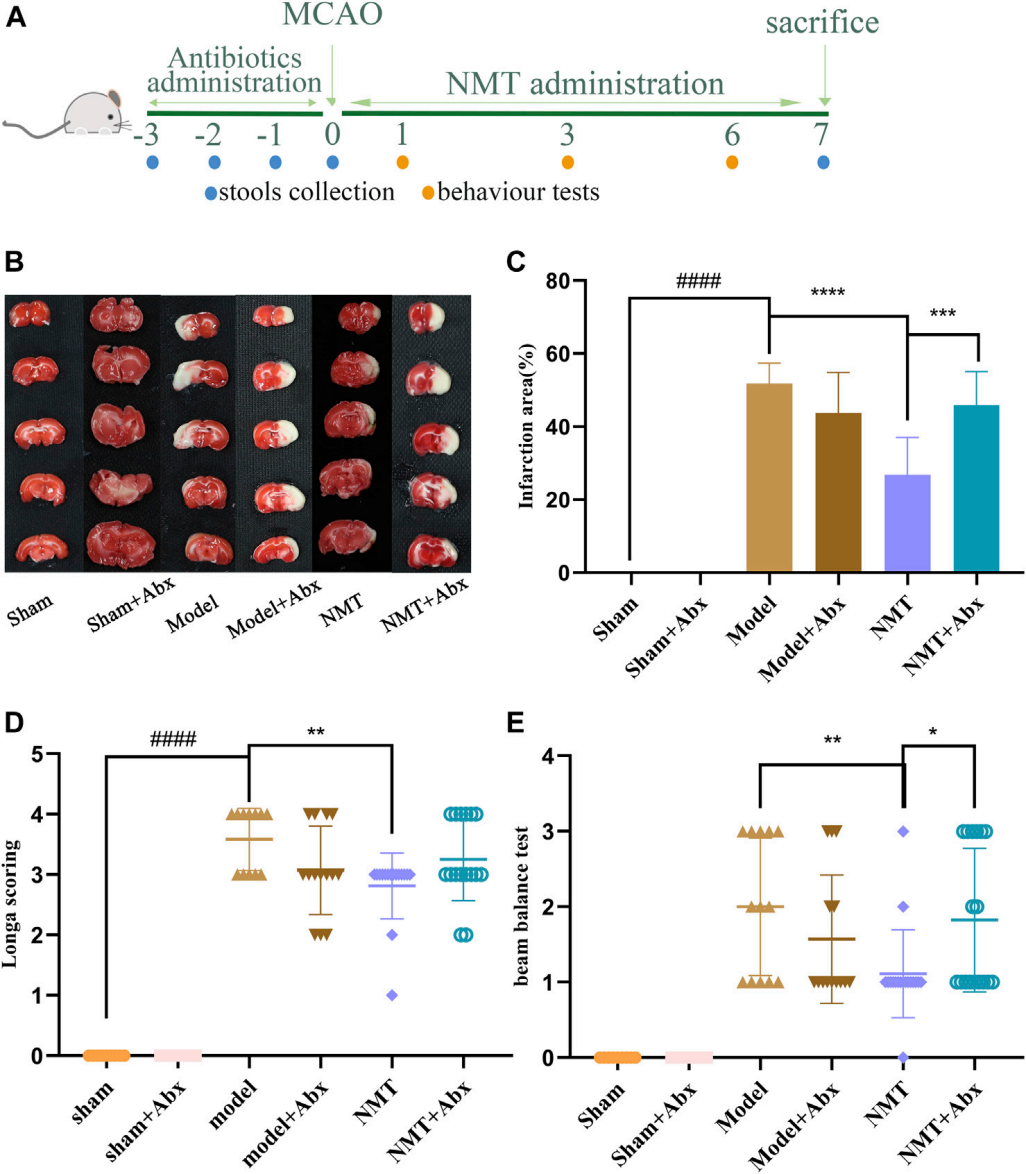
NMT depended on intestinal flora to improve stroke prognosis. **(A)** The procedures of animal experiments in PGF rats. **(B)** TTC staining. **(C)** Cerebral infarct area percentage (*n* = 6). Compared with sham group, statistical significance was considered at ##*p* < 0.01, ###*p* < 0.001 for all the test; Compared with model group, statistical significance was considered at *p < 0.05, **p < 0.01 and ***p < 0.001 for all the tests. **(D)** Neurological score (*n* = 9). **(E)** Beam balance test (*n* = 9). Error bars represent Mean ± SD.

To explore the efficacy of NMT, to reverse damage from ischemic stroke relies on the intestinal flora, PGF rats were used in the MCAO/R model. Firstly, the ability of Abx to decrease the abundance of microflora in stools was confirmed in oxygen-deficient conditions using a specific medium (Supplementary Figure S7). The efficacy of NMT was significantly lower in the absence of intestinal flora in terms of infarct area and neurological function scores. In the model and model plus Abx groups, the infarct area was 52.76 ± 5.24% and 43.76 ± 10.50%, respectively, indicating that excess antibiotics weakly reduced the effect of brain injury. Compared with model rats, administration of NMT reduced injury to 26.82 ± 9.34% while Abx rendered the benefit of NMT negligible (45.89 ± 8.37%). The NMT plus Abx treatment did not reduce infarct volume compared with NMT treatment (Figures 7B,C). Similarly, neurological function scores displayed the same trend (Figures 7D,E).

**FIGURE 8:**
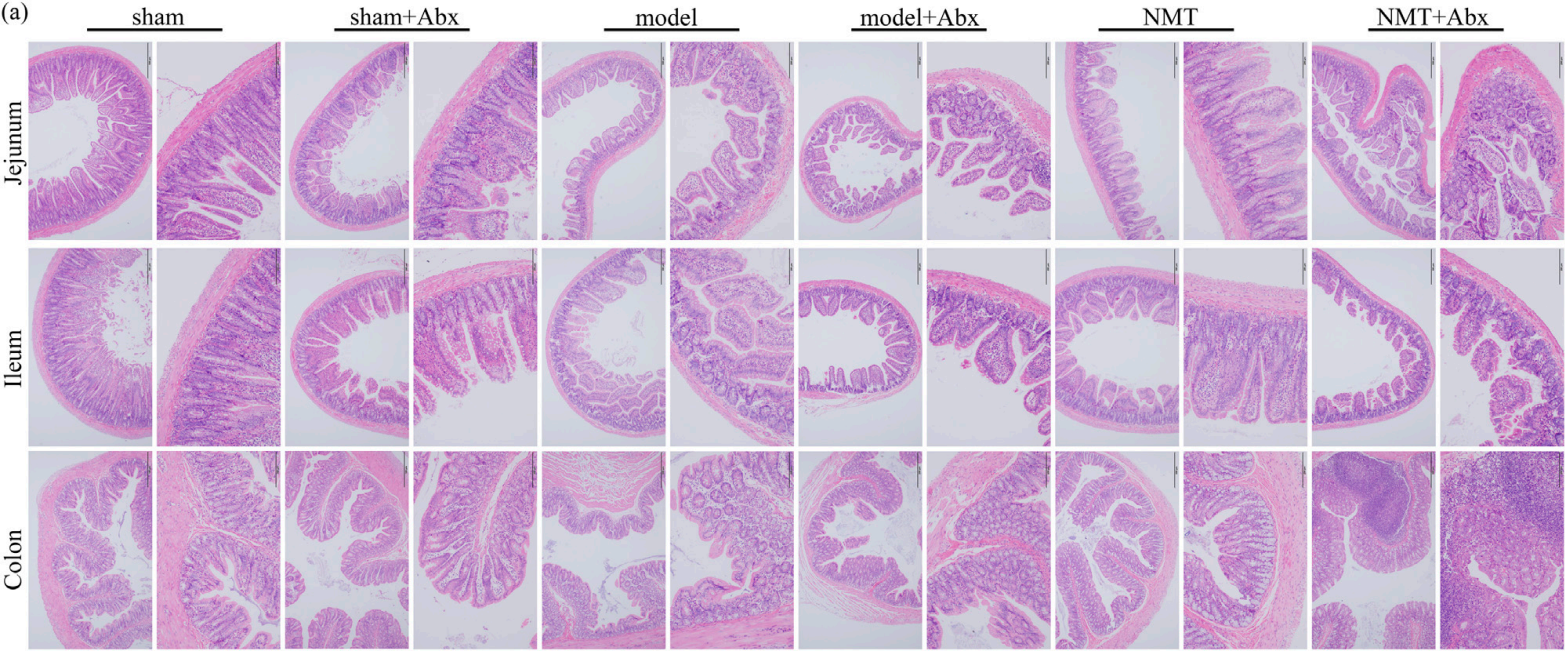
The lack of intestinal flora was not conducive to intestinal barrier repair. H&E Staining of jejunum, ileum and colon (scale bar = 200 and 100 μm *n* = 3).

**FIGURE 9:**
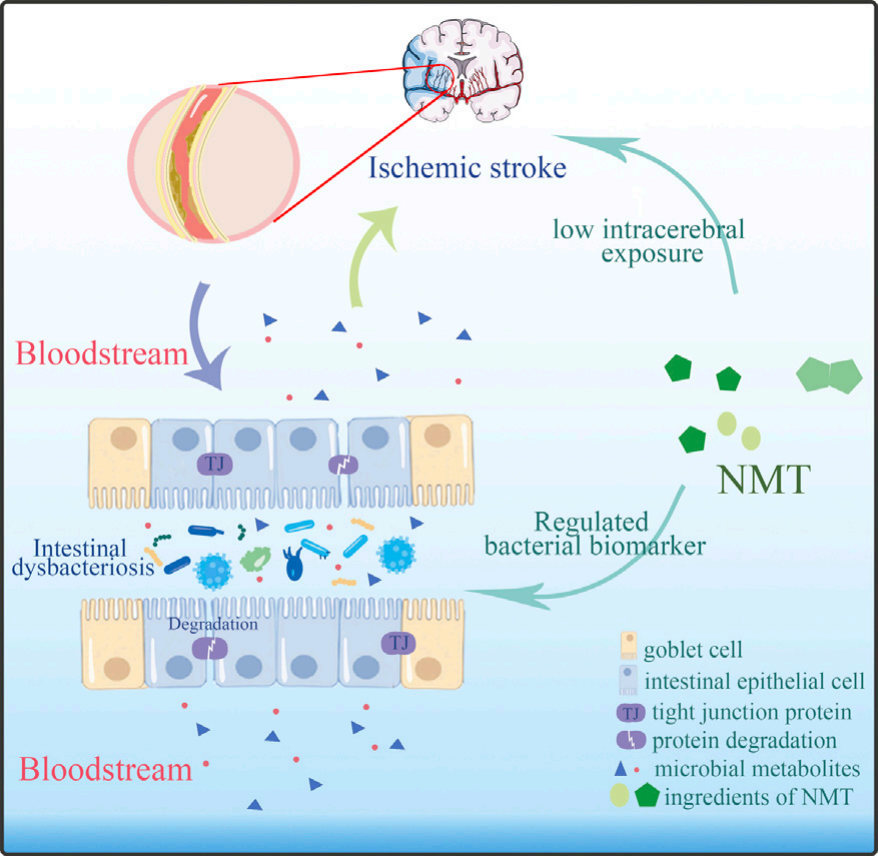
NMT modified the outcome of stroke by adjusted the imbalanced intestinal flora and its metabolic profiles.

The H&E staining indicated that tissue damage and infiltration by macrophages was distinctly reduced after the administration of NMT. More inflammatory cell infiltration was shown in the NMT plus Abx group indicating that the intestinal microbiota plays a significant role in reducing damage of intestinal tissue in stroke (Figure 8). These results revealed that the efficacy of NMT intervention in stroke relies on the gut microbiome (Figure 9).

## Discussion

Mounting evidences have revealed an association between microbial composition and their metabolites in IS. Recent studies had focused on cerebral ischemia with changes in intestinal microbiota community structure but ignored its connection with microbial metabolites. Thus, systematic investigation of the relationship between the gut microbiome and its metabolites is greatly warranted. We valued the changing gut microbiome and potential metabolites by NMT associated with the development and prognosis in cerebral ischemic stroke. We found that NMT modified the outcome of stroke in conventionalized (Conv) rats but not in PGF-rats. Total 35 differential bacterial genera were regulated by NMT. Furthermore, we confirmed 26 gut-derived differential metabolites, including lipids and lipid-like molecules, and organic acids and their derivatives which changing by NMT. Conspicuously, L-asparagine and indoleacetaldehyde were significantly negatively correlated with *Lachnospiraceae_UCG.001* and significantly positively correlated with *Lachnoclostridium*. Creatinine was strongly negatively correlated with Akkermansia. These associations highlighted potential interactions of microbe and intestinal microflora-derived metabolites during IS, which is helpful to further reveal the metabolic process *in vitro* during the development and prognosis of stroke.

Firstly, we identify several different genera in stroke outcomes treated by NMT. For example, the abundance of *Enterobacteriaceae* increased significantly in model rats, while *Ruminococcaceae* and *Lachnospiraceae* decreased in the prognosis of stroke (Figure 5, Supplementary Table S4). It had been reported that, following induction of cerebral ischemia, excessive nitrates form by free radical reactions and the expansion of *Enterobacteriaceae* exacerbated cerebral infarction by enhancing systemic inflammation (Xu et al., 2021). In the present study, NMT may repaired the intestinal barrier to promote stroke prognosis by inhibiting *Enterobacteriaceae*. Here, *Blautia,* a *Lachnospiraceae* family amino acid fermentation bacteria, increased in model rats, which may be related to protection from the host’s stress reaction, and increased continuously in the prognosis of stroke. *Blautia* was in high abundance during the recovery phase of the disease with an anti-inflammatory effect that improves recovery from pathogenic infection by regulating endogenous amino acid metabolism (Caballero et al., 2017; Hatziioanou et al., 2017; Kim et al., 2019). The present research indicated that the promotion of stroke prognosis may be related to the anti-inflammatory property of *Blautia*, while the specific anti-inflammatory mechanisms require further exploration. Moreover, the abundance of *Lactobacillus* increased significantly during the prognosis of stroke. The probiotic *Lactobacillus* resulted in increased levels of *p*-Akt and Bcl-2 in the hippocampus and decreased expression of Bax and Caspase-3 that reduces neuronal cell apoptosis in the hippocampal CA3 region (Xu et al., 2018). *Lactobacillus* catabolized tryptophan into indole and its derivatives in the stomach and ileum of mice (Zelante et al., 2013) which could reduce neuronal apoptosis (Tang et al., 2015; Luo et al., 2021). These may suggested that NMT reduced neuronal apoptosis by promoting the growth of *Lactobacillus* during the prognosis of stroke.

Interestingly, we found that the abundance of *Akkermansia,* a controversial bacterium, increased significantly in model rats and decreased during the prognosis of stroke (Figure 5, Supplementary Table S4). *Akkermansia* was able to degrade mucin prior to its utilization as a nutrient (Derrien, 2004) and secreted mucolytic enzymes such as glycosidases that eroded the mucus layer of the intestine which resulted in the infiltration of bacteria and endotoxins that exacerbated inflammation. Besides, it could also cause organ infection and lead to microorganism colonization in the intestinal mucosa that resulting in epithelial cell apoptosis (Benakis et al., 2016; Khan et al., 2020; Zhou et al., 2020). However, many studies had also reported that *Akkermansia* improved the inflammation and other adverse symptoms such as insulin resistance and glucose tolerance. It could also regulate the immune response and maintain metabolic balance (Jacobo de la Cuesta-Zuluaga, 2017; Grander et al., 2020; Rodriguez et al., 2020). The above results suggested that *Akkermansia* may cause the destruction of the intestinal barrier and translocation of bacteria, while the intestinal barrier was protected and inflammation was alleviated by inhibiting the growth of *Akkermansia* during the prognosis of stroke. The mechanism how *Akkermansia* influenced intestinal barrier function required additional exploration. Recently, *Roseburia*, a butyric acid-producing bacteria, was reported to possibly recover intestinal barrier integrity through recognition of Toll-like receptor 5 (TLR5) and upregulation of the tight junction protein Occludin, through augmentation of IL-22 and REG3g expression and restoration of the intestinal microbiota ecosystem (Kasahara et al., 2018; Zhuang et al., 2019; Liu et al., 2020). However, we found that it was not significantly upregulated in administrated rats but upregulated in model rats. Such inconsistent changes of gut microbiota and metabolites had been reported in many previous studies. There may be multiple reasons for these inconsistencies such as individual differences in animals, the different collection time of their fecal samples, their different rearing environment, or different software and supporting databases.

Furthermore, combined with untargeted metabolomic analysis, total 46 host different metabolites including 26 gut-derived metabolites changing by NMT during prognosis of IS. In the present study, a decrease in phenylacetylglycine (PAGly) during stroke correlated positively with levels of *Lachnospiraceae_UCG-001, Lachnospiraceae_UCG-006* and *Lactobacillus*, and correlated negatively with *Alistipes, Lachnoclostridium* and *Bacteroides* (Figure 6). Recent research indicated that incremental phenylacetylglutamine (PAGln) level was a dependable predictor of major detirmental cardiac events (Nemet et al., 2020). It was reported that the generation of PAGly (a metabolite in mice) or PAGln (a metabolite in humans) could be synthesized by phenylalanine in liver by combining with glutamine. PAGly and PAGln were metabolized to phenylacetic acid (Nemet et al., 2020) by the intestinal microbial *FldH* gene cluster in competition with the *porA* gene. Crucially, the balance between the relative activity of the enzymes *FldH* and *PorA* ultimately affected thrombosis in mice. Therefore, it was suggested that PAGln, a microbial metabolite found in *Christensenellaceae*, *Lachnospiraceae*, and *Ruminococcaceae*, may inhibit thrombosis by *FldH* and *PorA* gene. The researches on the current cases, PAGln could be a significant biomarker in stroke. Its metabolism and relationship with the gut microbiota in stroke will inevitably be the focus of future study.

Our research indicated that NMT reversed deoxycholic acid (DCA) level that was negatively correlated with (*Clostridium*)*_innocuum_group* and *Faecalitalea*, and positively correlated with (*Eubacterium*)*_nodatum_group*, *Candidatus_Saccharimonas*, and *Lachnospiraceae_UCG-006* (Figure 6). Bile acids (BA) are synthesized from cholesterol in the liver and most secreted into the intestinal lumen, where a substantial mass of toxic-hydrophobic bile acids was transformed, such as DCA (Nicholson et al., 2012; Doden et al., 2021). Recently, destruction of the intestinal barrier which correlated with secondary bile acids has been reported (Pi et al., 2020). The level of DCA was increased due to a significant increase in *Eubacterium* and expression of the enzyme encoding by bacterial gene such as 7α-dehydroxylation (*baij*). DCA down-regulated the expression of the ZO-1 and OCLD genes, which are associated with barrier function, while the expression of the EGFR and SRC genes was up-regulated. Consistently, we found that the change of DCA was positively correlated with the expression of ZO-1 (Figure 2E; Table 2). Thus, we consider that DCA is a potential drug target changed by NMT for intestinal barrier in IS.

In addition, fatty acids, had an ability to pass the blood-brain barrier, constituted up to 20% of total cerebral energy by its oxidation (Ebert et al., 2003). In the present study, higher proportions of palmitic acid, ricinoleic acid, and oleic acid were observed in MCAO rats, whereas a higher level of linoleic acid was observed after treatment. Linoleic acid (LA) supplementation can replace saturated fatty acid and monounsaturated fatty acids, possibly through multiple mechanisms such as decreasing platelet aggregation (Ristić-Medić et al., 2001; Luc et al., 2003) and blood pressure (Iacono et al., 1983; Al-Khudairy et al., 2015), crossing the blood-brain barrier to furnish energy to the cerebrum (Panov et al., 2014), assisting the deformability of erythrocytes against stroke (Tsai et al., 2012; Veno et al., 2017). The results indicated that higher proportions of palmitic acid, ricinoleic acid, and oleic acid caused an increasing risk of stroke by disruption of the intestinal barrier and facilitation of bacterial migration, whereas higher levels of linoleic acid against stroke by providing of energy (Bretagne et al., 1981; Morehouse et al., 1986; Wiberg et al., 2006). In addition, we found that phosphatidylcholine (PC), arachidonic acid (AA), and sphingomyelin (SM) were declined significantly in model rats but not in the NMT group (Table 2). AA, a ubiquitous membrane constituent, was degraded from PC and phosphatidylethanolamine (PE) by various lipases, such as monoacylglycerol lipase (MAGL), phospholipase C (PLC) and phospholipase A2 (PLA2) in cell membranes (Rink and Khanna, 2011; Choi et al., 2018). Arachidonic acid was converted by cyclooxygenase and lipoxygenase, producing inflammatory factors such as prostaglandins, leukotrienes, and thromboxane that induced inflammation and additional neuronal apoptosis (Rink and Khanna, 2011). The results here indicated that severe inflammation and neural apoptosis were associated with decreased levels of AA, PC, and SM, which in turn were associated with decreased levels of *Bacteroides* (*Clostridium*)*_innocuum_group,* and increased levels of *Candidatus_Saccharimonas* and *Lachnospiraceae_UCG-006* (Figure 6, Figure 7)*.*

Enriching and analyzing the different metabolites, IS will cause dysbiosis of metabolism particularly linoleic acid metabolism, arginine and proline metabolism, aminoacyl-tRNA biosynthesis, and propanoate metabolism. The disorder of linoleic acid metabolism in the model rats indicated the aggravation of stroke. ALDH4A1 reduced the free cholesterol and low density lipoprotein (LDL) to improve atherosclerosis progression by involving in proline metabolism (Lorenzo et al., 2021). Arginine metabolism was closely related the activity of nitric oxide synthases (NOS) and cyclooxygenases (COX) which were associated to neuroprotection (Nair et al., 2011). The disorder of arginine and proline metabolism may exacerbate IS. Aminoacyl-tRNA was an essential substrate to transport homologous amino acid to the extending polypeptide chain (Sheppard et al., 2008; Yao and Fox, 2013). Dysregulation of aminoacyl-tRNA biosynthesis by IS may affect the function of protein synthesis. After the treatment of NMT, various metabolic pathways were regulated, including citrate cycle (TCA cycle), glycine, serine and threonine metabolism and phenylalanine metabolism. Cystine protected brain cells from the toxic effects of zinc which released from damaged neurons in IS by chelating zinc or transforming to tricarboxylic acid cycle intermediates (Ralph et al., 2010). TCA cycle produced more than 90% of the cellular energy (Pieczenik and Neustadt, 2007) which is necessary to the activity of neuronal cells. The disturbance of TCA cycle aggravated neuronal damage and irreversible brain injury (Mccall, 2004). Consequently, NMT may provide enough energy and protect neurons by adjusted glycine metabolism and TCA cycle after IS.

Here, we focused on the relationship between the microbiota with significant change in genus level and its metabolites during stroke recovery treated by NMT. Although our data do not point to an underlying biological mechanism, they focus future experiments on investigating candidate pathways that relate to 26 gut-derived metabolites. Our research contributed to the characterization of biochemical processes in ischemic brain tissue or the discovery of different chemical reactions that control the development of stroke. In subsequent studies, the specific relationship between the key bacterial genera and the differential metabolites, as well as their relationship with stroke-related pathological mechanisms such as oxidative stress, immune response and nerve damage, will be the focus of stroke prognosis research, especially the relationship between L-Asparagine, Indoleacetaldehyde, N-Formyl-L-aspartate and *Lactobacillaceae, Akkermansiaceae, Lachnospiraceae, Ruminococcaceae*.

## Conclusion

The present research explored the relationship between gut microbiota and endogenous metabolites in IS treated by NMT using 16S rRNA gene sequencing and metabonomic analysis. Total 35 differential bacterial genera and 26 gut-derived differential metabolites were confirmed. We identified the connection between intestinal microflora and their metabolites in IS treated by NMT. Especially, L-asparagine and indoleacetaldehyde were significantly negatively correlated with *Lachnospiraceae_UCG.001* and significantly positively correlated with *Lachnoclostridium*. Indoleacetaldehyde also presented the negative correlation with *Lactobacillus* and *Bifidobacterium*. 2-Hydroxybutyric acid was strongly negatively correlated with *Ruminococcus, Lachnospiraceae_UCG.001* and *Lachnospiraceae_UCG.006.* Creatinine was strongly negatively correlated with *Akkermansia*. The critical relationships of our research would provide evidence for the interpretation of mechanism in IS and the use of exogenous probiotics or prebiotics in clinical application.

## Data Availability

The datasets presented in this study can be found in online repositories. The names of the repository/repositories and accession number(s) can be found below: https://www.ncbi.nlm.nih.gov/bioproject/, accession ID: PRJNA769802.
