# Supplementary material for: Integrated Analysis of the Cecal Microbiome and Plasma Metabolomics to Explore NaoMaiTong and Its Potential Role in Changing the Intestinal Flora and Their Metabolites in Ischemic Stroke
Source: Front Pharmacol. 2022 Jan 20;12:773722. doi: 10.3389/fphar.2021.773722 (PMC8811223; doi:10.3389/fphar.2021.773722)
Supplement: Supplementary file 1 [file DataSheet1.docx]

Supplementary Material

# Supplementary Figures and Tables


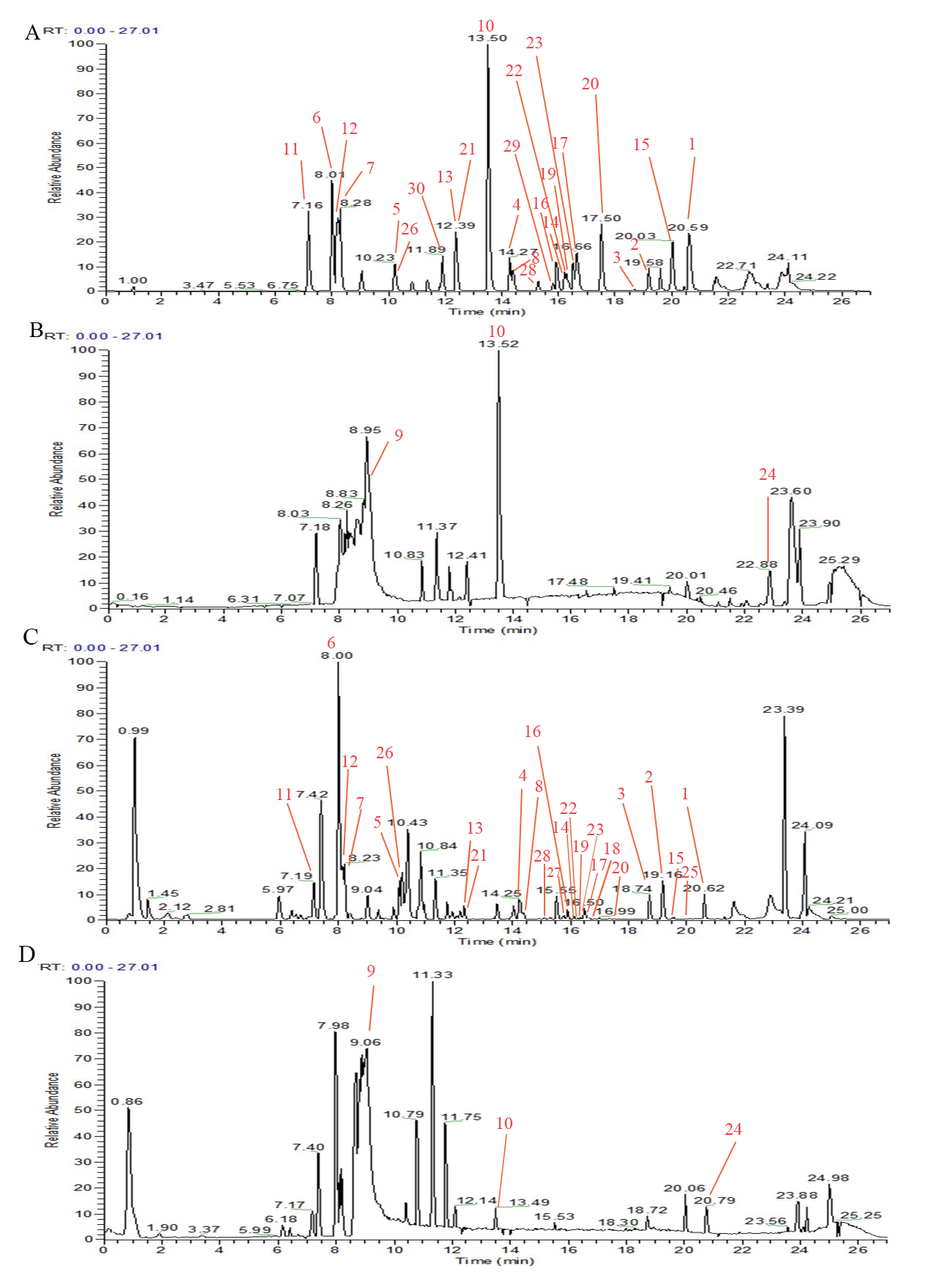


**Supplementary Figure 1┃**Total ion chromatograms of a mixture of multi-standards obtained from the negative (A) and positive (B) ion scan modes. Total ion currents (TIC) chromatograms of the major constituents in NMT decoction. The compounds are detected under negative (C) and positive (D) mode, respectively.

**Supplementary Table 1┃**The main components from NMT .

| Number | Constituents | RT (min) | Molecular formula | Measured value (m/z) |
| --- | --- | --- | --- | --- |
| 1 | Emodin | 20.63 | C15 H10 O5 | 269.04559 [M-H]- |
| 2 | Rhein | 19.22 | C15H8O6 | 283.02487 [M-H]- |
| 3 | Aloe-Emodin | 18.69 | C15 H10 O5 | 269.04572 [M-H]- |
| 4 | Emodin-8-glucoside | 14.33 | C21H20O10 | 431.09851 [M-H]- |
| 5 | Rhein-8-glucoside | 10.26 | C21H18O11 | 445.07791 [M-H]- |
| 6 | Puerarin | 8.1 | C21H20O9 | 415.10358 [M-H]- |
| 7 | 3''-Methyoxy puerarin | 8.35 | C22H22O10 | 445.11423 [M-H]- |
| 8 | Chrysophal 8-O-glucoside | 14.47 | C21 H20 O9 | 415.10367 [M-H]- |
| 9 | Daidzin | 9.07 | C21 H20 O9 | 417.11804 [M+H]+ |
| 10 | Daidzein | 13.55 | C15H10O4 | 255.06519 [M+H]+ |
| 11 | 3'-Hydroxy Puerarin | 7.24 | C21H20O10 | 431.09879 [M-H]- |
| 12 | Mirificin | 8.26 | C26H28O13 | 547.146 [M-H]- |
| 13 | Ginsenoside Rg1 | 12.46 | C42 H72 O14 | 845.49103 [M+HCOO]- |
| 14 | Ginsenoside Rg2 | 16.28 | C42 H72 O13 | 829.49664 [M+HCOO]- |
| 15 | Ginsenoside Rg3 | 19.99 | C42 H72 O13 | 829.49719 [M+HCOO]- |
| 16 | Ginsenoside Rb1 | 16.00 | C54H92O23 | 1153.60181 [M+HCOO]- |
| 17 | Ginsenoside Rb2 | 16.76 | C54 H90O24 | 1123.59143 [M-H]- |
| 18 | Ginsenoside Rb3 | 16.85 | C53H90O22 | 1123.5918 [M-H]- |
| 19 | Ginsenoside Rc | 16.37 | C53H90O22 | 1123.59241 [M+HCOO]- |
| 20 | Ginsenoside Rd | 17.61 | C48 H82 O18 | 991.54889 [M+HCOO]- |
| 21 | Ginsenoside Re | 12.46 | C48 H82 O18 | 991.54889 [M+HCOO]- |
| 22 | Ginsenoside Ra1 | 16.34 | C58H98O26 | 1255.63403 [M+HCOO]- |
| 23 | Ginsenoside Ro | 16.62 | C48 H76O19 | 955.492 [M-H]- |
| 24 | Ligustilide | 20.81 | C12 H14 O2 | 191.10672 [M+H]+ |
| 25 | Senkyunolide A | 20.09 | C12H16O2 | 193.12234 [M+H]+ |
| 26 | ferulic acid | 10.34 | C10H10O4 | 193.05011 [M-H]- |
| 27 | Ginsenoside Ra2 | 15.83 | C58H98O26 | 1255.63403 [M+HCOO]- |
| 28 | Ginsenoside Rf | 15.38 | C42 H72 O14 | 845.4917 [M+HCOO]- |
| 29 | Emodin methyl ether-8-O glucoside | 15.83 | C22H22O10 | 445.11456 [M-H]- |
| 30 | notoginsenoside R1 | 11.93 | C47 H80 O18 | 977.53351 [M+HCOO]- |

**Supplementary Table 2┃**The standards of neurological function score.

| Score | Symptom |
| --- | --- |
| 0 | no neurologic deficit |
| 1 | failure to extend left forepaw fully: a mild focal neurologic deficit |
| 2 | circling to the left: a moderate focal neurologic deficit |
| 3 | falling to the left: a severe focal deficit |
| 4 | did not walk spontaneously and had a depressed level of consciousness |

**Supplementary Table 3┃**The standards of beam balance test.

| Score | Symptom |
| --- | --- |
| 0 | the four limbs were all on the wood in a balance situation |
| 1 | the limbs of one side were able to grasp the wood or shake on the wood |
| 2 | one or two limbs slipped from the wood |
| 3 | three limbs slipped from the wood |
| 4 | the rat was suspended on the wood and fell over after a struggle |


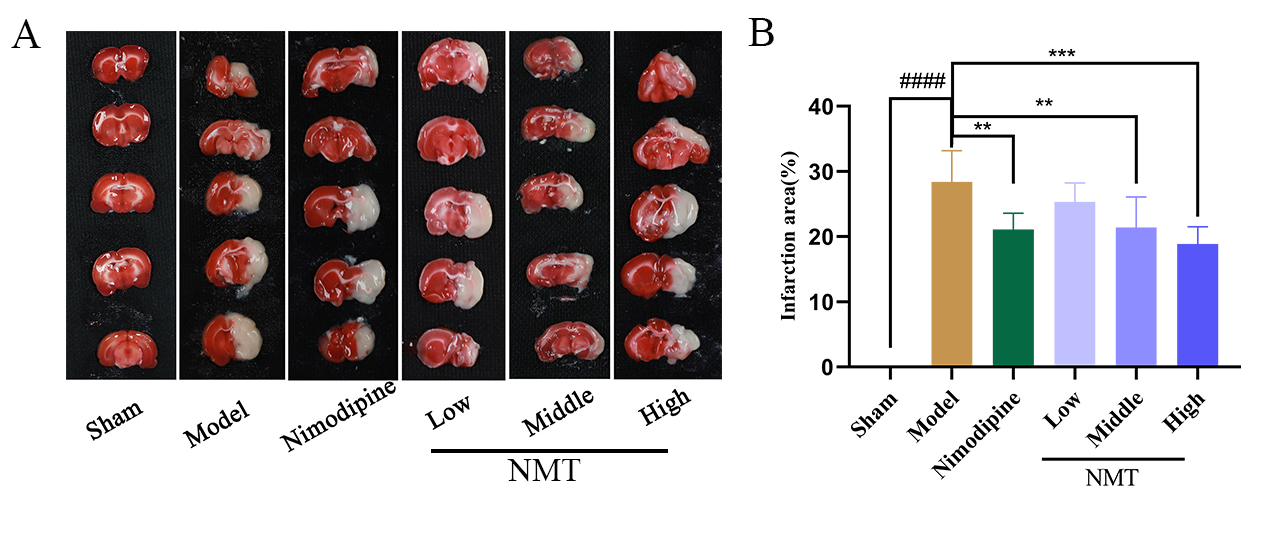


**Supplementary Figure 2┃Middle, high doses of NMT reduced infract volume after MCAO in rats.** (A) TTC staining. (B) Cerebral infarct area percentage (n > = 6). The comparison was analyzed ordinary one-way ANOVA. Compared with sham group, statistical significance was considered at ###p < 0.001 for all the test; Compared with model group, statistical significance was considered at *p < 0.05, **p < 0.01 and ***p < 0.001 for all the tests.


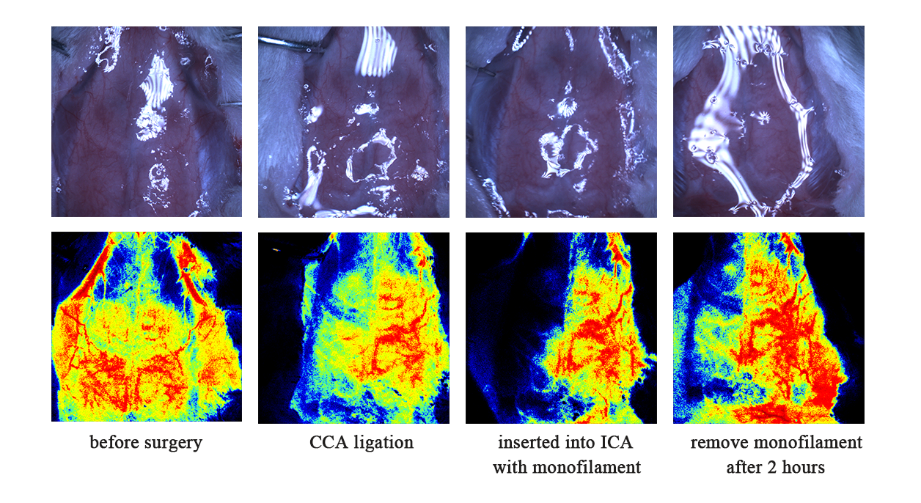


**Supplementary Figure 3┃The changes of cerebral blood flow (CBF) during the process of surgery.** The CBF was measured by Laser Speckle Flowgraphy system in before surgery, the ligation of right common carotid artery (CCA), the monofilament insertion of Internal carotid artery (ICA), the remove of monofilament after 2 hours.


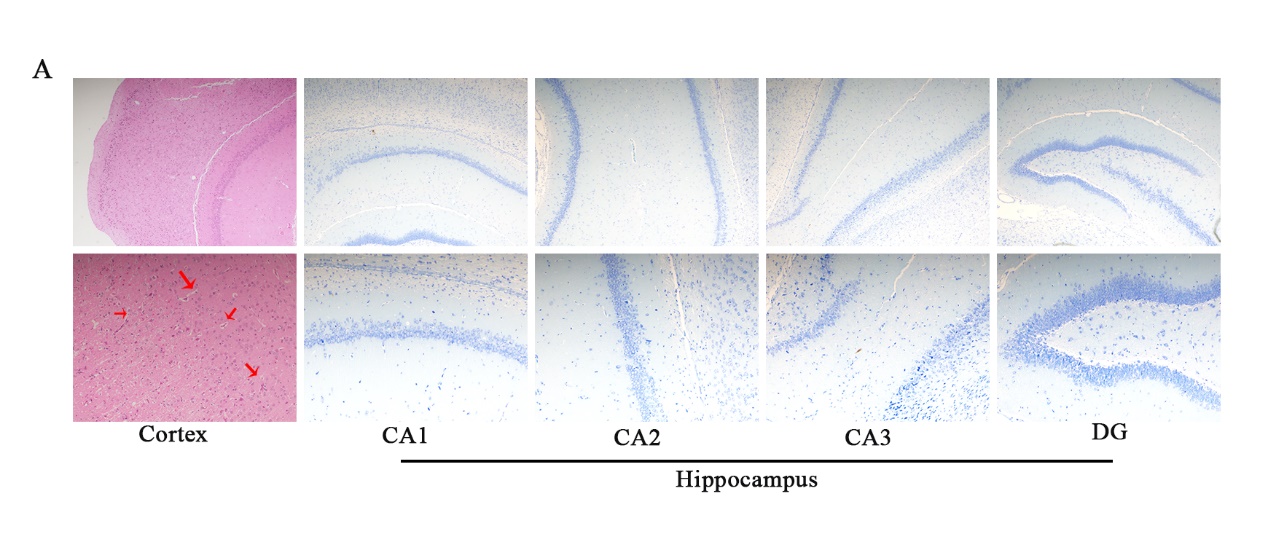


**Supplementary Figure 4┃****The pathological section of cerebral cortex and cerebral hippocampus in Nimodipine treatment group.** H&E Staining of cerebral cortex and Nissl's staining of cerebral hippocampus difference partition such as CA1, CA2, CA3, DG in Nimodipine treatment group. The red arrows represent apoptotic neurons.


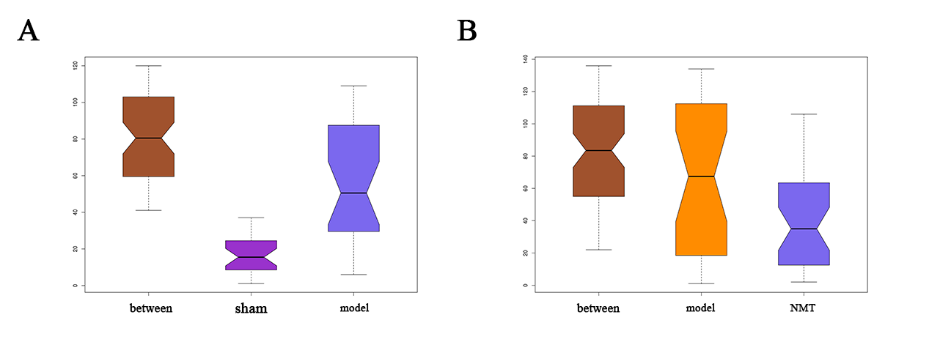


| Group | R-value | *p*-value |
| --- | --- | --- |
| sham-model | 0.7405 | 0.001 |
| NMT-model | 0.4635 | 0.001 |

**Supplementary Figure 5┃The Anosim analysis between different groups.** (A) The Anosim analysis of sham and model groups; (B) The Anosim analysis of model and NMT groups; R-value is between (-1, 1). If R-value is greater than 0, the difference between groups is significant. If R-value is less than 0, the intra-group difference is greater than the inter-group difference. *p* < 0.05 indicated statistical significance.


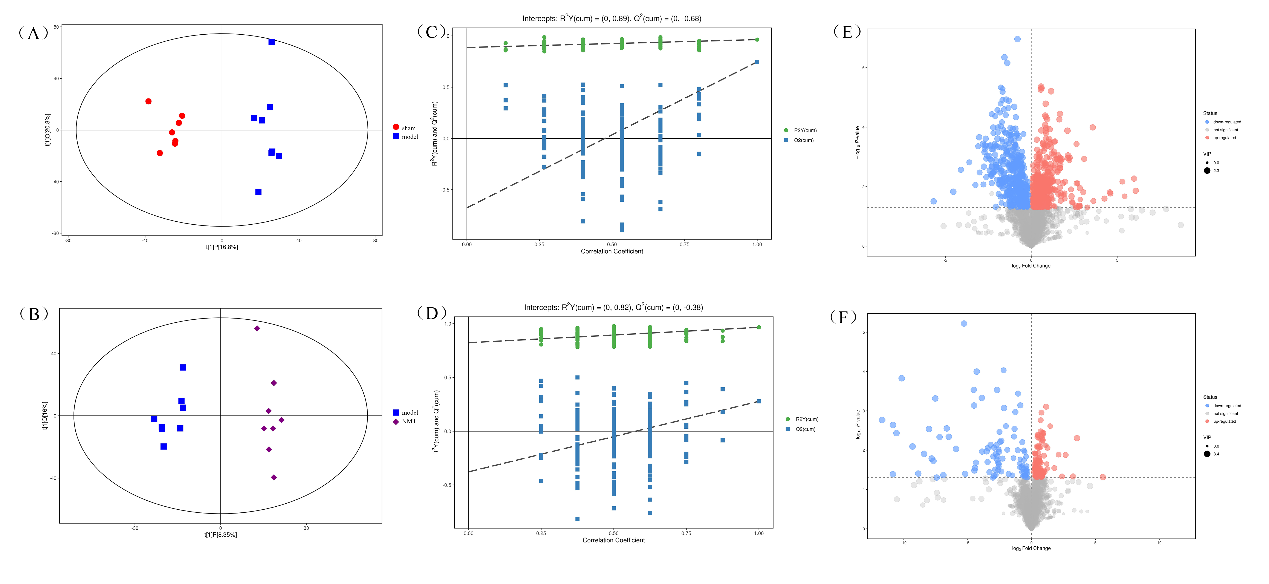


**Supplementary Figure 6┃The significant changes in metabolic profiles of gut microbiota in stroke and recovered rats**. (A) The plots of OPLS-DA scores of all peak features in positive (ES+) ion modes from the untargeted metabolomics analysis of stool samples of the rats in sham (n = 7) and model (n = 8) groups; (B) The plots of OPLS-DA scores of all peak features in positive (ES+) ion modes of the rats in model (n = 8) and NMT (n = 8) groups; (C) Positive scatter plots of the statistical validations obtained by 200X permutation tests in sham (n = 7) and model (n = 8) groups. R2 measures the goodness of fit and Q2 measures the predictive ability of the model. The criterion for model validity is that the regression line of the Q2-points (blue dotted line) intersects the vertical solid line (on the left) below zero; (D) Positive scatter plots of in model (n = 8) and NMT (n = 8) groups; (E) Positive volcano plot; volcano plots of the peak features of intestinal metabolites which significantly changed in three group in positive ion mode (ES+, upper panel). Red and blue circles indicate the significantly increased and decreased metabolites, respectively, (VIP > 1, *p* < 0.05) in model VS sham group. The color tone indicates *p* value: a dark color indicates a small *p* value. The circle radius indicates the VIP value of corresponding peak features. (F) Positive volcano plot in model vs. NMT group.

**Supplementary Table 4┃**Effect of NaoMaiTong (NMT) on families of bacteria in the microbiome of MCAO/R male rats.

| Taxon | group | | |
| --- | --- | --- | --- |
|  | Sham (n = 8) | Model (n = 8) | NMT (n = 9) |
| *Lachnospiraceae_NK4A136_group* | 0.0756±0.031 | 0.0528±0.0779 | 0.0381±0.0171 |
| *Colidextribacter* | 0.0321±0.0154 | 0.0163±0.0155 | 0.0134±0.0101 |
| *Dubosiella* | 0.0008±0.0006 | 0.0043±0.0054 | 0.0012±0.0011 |
| *Bacteroides* | 0.0053±0.0025 | 0.0571±0.0569 | 0.028±0.0175 |
| *Escherichia-Shigella* | 0.0015±0.0019 | 0.0793±0.1188 | 0.0143±0.0175 |
| *Alloprevotella* | 0.0065±0.0101 | 0.0017±0.0019 | 0.0021±0.0023 |
| *[Eubacterium]_siraeum_group* | 0.0008±0.0007 | 0.0006±0.0003 | 0.0027±0.006 |
| *Akkermansia* | 0.0016±0.0017 | 0.1362±0.1899 | 0.0058±0.0074 |
| *Clostridium_sensu_stricto_1* | 0.021±0.0133 | 0.0044±0.0018 | 0.0079±0.0063 |
| *Allobaculum* | 0.0011±0.001 | 0.0362±0.0466 | 0.0034±0.0065 |
| *Coprococcus* | 0.001±0.0008 | 0.0055±0.0046 | 0.0138±0.0114 |
| *Faecalitalea* | 0.0001±0.0001 | 0.0132±0.0326 | 0.0053±0.0119 |
| *Alistipes* | 0.0081±0.015 | 0.0014±0.0009 | 0.0034±0.0013 |
| *Prevotellaceae_NK3B31_group* | 0.0002±0.0003 | 0.0015±0.0017 | 0.0031±0.004 |
| *Desulfovibrio* | 0.005±0.0019 | 0.0051±0.0045 | 0.0064±0.0029 |
| *Parabacteroides* | 0.0011±0.0011 | 0.0028±0.0027 | 0.0047±0.0044 |
| *NK4A214_group* | 0.0089±0.0047 | 0.0036±0.0019 | 0.0048±0.0018 |
| *Romboutsia* | 0.0403±0.0248 | 0.0165±0.0086 | 0.0183±0.0136 |
| *Blautia* | 0.007±0.0081 | 0.0298±0.0212 | 0.0484±0.0272 |
| *Roseburia* | 0.027±0.0143 | 0.0716±0.0784 | 0.0116±0.0074 |
| *Lachnoclostridium* | 0.0012±0.0006 | 0.0141±0.0154 | 0.0115±0.0093 |
| *Ruminococcus* | 0.0075±0.0032 | 0.0063±0.0029 | 0.0148±0.007 |
| *Marvinbryantia* | 0.0019±0.0018 | 0.0059±0.0038 | 0.0254±0.0285 |
| *[Eubacterium]_xylanophilum_group* | 0.0153±0.0176 | 0.0021±0.0021 | 0.0039±0.0025 |
| *[Ruminococcus]_gnavus_group* | 0.0036±0.0021 | 0.0105±0.0105 | 0.0244±0.0281 |
| *Lachnospiraceae_NK4B4_group* | 0.0012±0.0013 | 0.0025±0.0028 | 0.0149±0.0122 |
| *Lactobacillus* | 0.2538±0.1041 | 0.0611±0.051 | 0.0857±0.0983 |
| *Turicibacter* | 0.0139±0.0075 | 0.0044±0.0023 | 0.016±0.0155 |
| *UCG-005* | 0.0012±0.0011 | 0.0077±0.009 | 0.0222±0.0274 |
| *Bifidobacterium* | 0.0153±0.0161 | 0.0005±0.0003 | 0.0011±0.0019 |
| *Parasutterella* | 0.0113±0.0052 | 0.0086±0.0038 | 0.0231±0.0143 |
| *Prevotellaceae_UCG-001* | 0.0023±0.0019 | 0.0168±0.0255 | 0.0039±0.0042 |
| *Candidatus_Saccharimonas* | 0.0087±0.0049 | 0±0 | 0.0005±0.0013 |
| *Monoglobus* | 0.0032±0.0017 | 0.0018±0.001 | 0.0099±0.0121 |
| *Oscillibacter* | 0.0159±0.0152 | 0.0032±0.003 | 0.0042±0.004 |

**Supplementary Table 5┃**The pathway and relative abundance of sham/model group represented by KO in Fig5 e.

| Taxa | pathway name | avg(sham) | avg(model) | *p*.value | q.values |
| --- | --- | --- | --- | --- | --- |
| K02025 | multiple sugar transport system permease protein | 0.009372205 | 0.00497069 | 0.004150219 | 0.019449765 |
| K02026 | multiple sugar transport system permease protein | 0.008771586 | 0.004630528 | 0.003447761 | 0.016887437 |
| K02027 | multiple sugar transport system substrate-binding protein | 0.005092577 | 0.002957489 | 0.007416894 | 0.028975644 |
| K09687 | antibiotic transport system ATP-binding protein | 0.003764973 | 0.003163421 | 0.038113238 | 0.075499583 |
| K01990 | ABC-2 type transport system ATP-binding protein | 0.003321657 | 0.003004067 | 0.007398986 | 0.02895535 |
| K07024 | sucrose-6-phosphatase | 0.003259097 | 0.002663096 | 0.045363037 | 0.082191365 |
| K02006 | cobalt/nickel transport system ATP-binding protein | 0.00265726 | 0.001916836 | 0.008738928 | 0.032358912 |
| K01834 | 2,3-bisphosphoglycerate-dependent phosphoglycerate mutase | 0.002615937 | 0.002145546 | 0.02957525 | 0.065572018 |
| K02035 | peptide/nickel transport system substrate-binding protein | 0.002413913 | 0.001911155 | 1.90E-06 | 0.000150055 |
| K09686 | antibiotic transport system permease protein | 0.001961936 | 0.001767623 | 0.038678956 | 0.075830421 |
| K02034 | peptide/nickel transport system permease protein | 0.001946472 | 0.00168237 | 0.013664945 | 0.044589442 |
| K00974 | tRNA nucleotidyltransferase (CCA-adding enzyme) | 0.001854209 | 0.001697492 | 0.031227903 | 0.067516017 |

**Supplementary Table 6┃**The pathway names and relative abundance of model/NMT group represented by KO in Fig5 f.

| Taxa | pathway name | avg(model) | avg(NMT) | *p*.value | q.values |
| --- | --- | --- | --- | --- | --- |
| K02025 | multiple sugar transport system permease protein | 0.00497069 | 0.006927265 | 0.049362012 | 0.080323382 |
| K02026 | multiple sugar transport system permease protein | 0.004630528 | 0.006494246 | 0.04286357 | 0.074354768 |
| K02027 | multiple sugar transport system substrate-binding protein | 0.002957489 | 0.004131893 | 0.043092436 | 0.074621775 |
| K09687 | antibiotic transport system ATP-binding protein | 0.003163421 | 0.004201745 | 0.001992443 | 0.032043442 |
| K01990 | ABC-2 type transport system ATP-binding protein | 0.003004067 | 0.003324342 | 0.00997256 | 0.038702004 |
| K02006 | cobalt/nickel transport system ATP-binding protein | 0.001916836 | 0.002527885 | 0.022730346 | 0.051327238 |
| K03091 | RNA polymerase sporulation-specific sigma factor | 0.001719211 | 0.002270122 | 0.044884356 | 0.076135863 |
| K09686 | 2,3-bisphosphoglycerate- antibiotic transport system permease protein | 0.001767623 | 0.002118187 | 0.000391242 | 0.032043442 |
| K00850 | 6-phosphofructokinase | 0.001759267 | 0.002016021 | 0.022272221 | 0.050573883 |
| K06180 | ribosomal large subunit pseudouridine synthase D | 0.001682529 | 0.002002452 | 0.003235173 | 0.032043442 |
| K01534 | Cd2+/Zn2+-exporting ATPase | 0.001718233 | 0.00198617 | 0.026864163 | 0.057045097 |
| K02033 | peptide/nickel transport system permease protein | 0.001596187 | 0.001787897 | 0.035566335 | 0.067300107 |


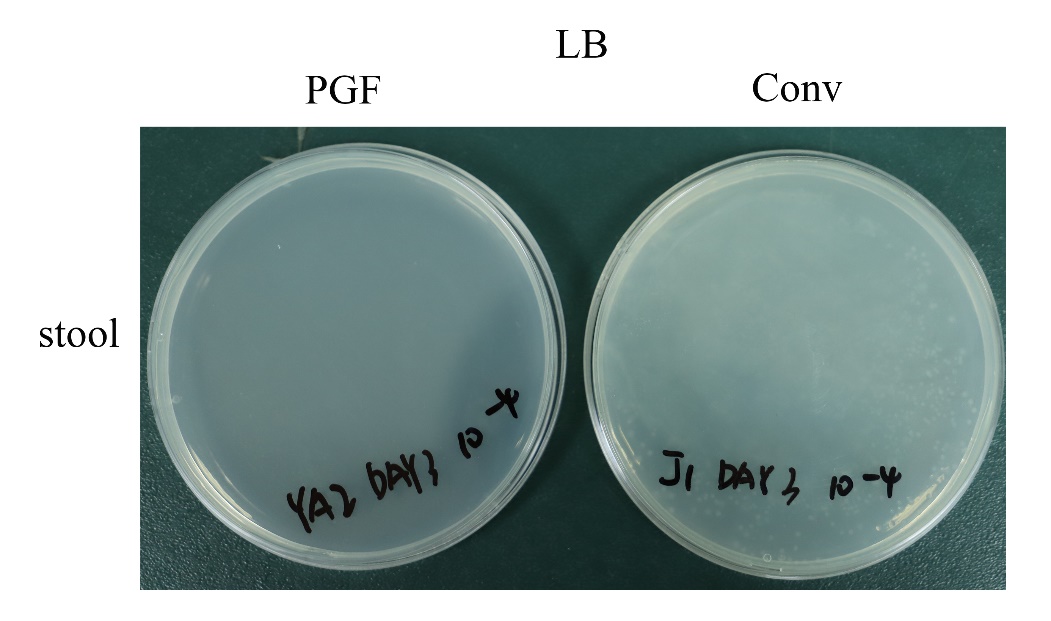


**Supplementary Figure 7┃Bacterial growth results for fecal samples of PGF model rats.** The fecal of all PGF rats were verified and representative photographs are shown.
